# Observation of the Same New Sheet Topology in Both the Layered Uranyl Oxide-Phosphate Cs_11_[(UO_2_)_12_(PO_4_)_3_O_13_] and the Layered Uranyl Oxyfluoride-Phosphate Rb_11_[(UO_2_)_12_(PO_4_)_3_O_12_F_2_] Prepared by Flux Crystal Growth

**DOI:** 10.3389/fchem.2019.00583

**Published:** 2019-08-21

**Authors:** Christian A. Juillerat, Vancho Kocevski, Theodore M. Besmann, Hans-Conrad zur Loye

**Affiliations:** ^1^Department of Chemistry and Biochemistry, University of South Carolina, Columbia, SC, United States; ^2^Center for Hierarchical Wasteform Materials (CHWM), University of South Carolina, Columbia, SC, United States; ^3^Nuclear Engineering Program, University of South Carolina, Columbia, SC, United States

**Keywords:** flux crystal growth, uranyl, phosphuranylite, single crystal, DFT

## Abstract

Single crystals of four new layered uranyl phosphates, including three oxyfluoride-phosphates, were synthesized by molten flux methods using alkali chloride melts, and their structures were determined by single-crystal X-ray diffraction. Cs_11_[(UO_2_)_12_(PO_4_)_3_O_13_] (**1**) and Rb_11_[UO_2_)_12_(PO_4_)_3_O_12_F_2_] (**2**) contain uranyl phosphate layers exhibiting a new sheet topology that can be related to that of β-U_3_O_8_, while Cs_4.4_K_0.6_[(UO_2_)_6_O_4_F(PO_4_)_4_(UO_2_)] (**3**) and Rb_4.4_K_0.6_[(UO_2_)_6_O_4_F(PO_4_)_4_(UO_2_)] (**4**) contain layers of a known isomer of the prominent phosphuranylite topology. The location of the fluorine in structures **2**-**4** is discussed using bond valence sums. First principles calculations were used to explore why a pure oxide structure is obtained for the Cs containing phase (**1**) and in contrast an oxyfluoride phase for the Rb containing phase (**2**). Ion exchange experiments were performed on **1** and **2** and demonstrate the ability of these structures to exchange approximately half of the parent alkali cation with a target alkali cation in an aqueous concentrated salt solution. Optical measurements were performed on **1** and **2** and the UV-vis and fluorescence spectra show features characteristic of the ${\text{UO}}_{2}^{2+}$ uranyl group.

## Introduction

Nuclear power has been well-established for several decades and, nonetheless, studies continue to develop a deeper understanding of the nuclear fuel cycle, including exploring improved methods of both long-term and short-term waste storage (zur Loye et al., 2018), and continuing to investigate the processes of radionuclide leaching into surrounding ecosystems. For these reasons, it is advantageous to further expand our understanding of uranium coordination chemistry, specifically in extended structures, as this can give us insights in understanding intermediate phases in the nuclear waste cycle, identifying potential structures useful in nuclear waste processing or storage, and possible pathways within the environment for the migration of U^6+^ and other actinides.

Nature often gives a good indication of what chemical compounds can be made synthetically. For example, uranium containing minerals present a few prominent sheet anion topologies that can be observed both in minerals and synthetic compounds. In Lussier et al. (2016) most recent review of hexavalent uranium compounds, autunite, phosphuranylite, and uranophane are significant minerals classes with 40 proposed autunite minerals, 16 phosphuranylite, and 10 uranophane minerals. Phosphuranylite and autunite minerals are the primary classes of phosphorus containing minerals, as phosphorus bearing uranium minerals make up nearly a quarter of all identified uranium minerals. The uranophane topology is specifically prominent among silicates, but as in the phosphuranylite topology the tetrahedrally coordinated Si or P sites can be replaced by other tetrahedrally—or even trigonal pyramidal or trigonal planar—coordinating elements. These sheet anion-topologies common among minerals have also been observed in numerous synthetic compounds including 38 belonging to the autunite, 18 belonging to the uranophane, and 16 belonging to the phosphuranylite classes (Lussier et al., 2016; Wang et al., 2017; Juillerat et al., 2018a;Juillerat and zur Loye, 2019).

While nature certainly gives a good indication of what we might be able to synthesize in the laboratory, many additional sheet topologies outside of those found in minerals are also reported [62 reported in Lussier et al. (2016)]. All of the discussed sheet topologies so far have been for uranium oxide compounds and one way to expand the number of known sheet topologies, and thus our understanding of uranium chemistry, is to partially exchange oxygen within these sheets with other anions such as a halides or sulfides, although in this paper we will only discuss halides. While one may expect to make radically different sheet structures using Cl^−^ due to the commonly terminal nature and large size of this anion, causing it to stick out of the plane of the sheet, as seen in K_4_U_5_O_16_Cl_2_ and Cs_5_U_7_O_22_Cl_3_ (Read et al., 2014), the inclusion of F^−^ could lead to new sheet structures or to those already observed in oxides. This arises partially due to the similarity in size of O and F, and examples can be seen in the existence of both rare earth oxides and oxyfluorides that adopt the prominent apatite structure (Latshaw et al., 2014, 2015). In rare earth silicates, the coordination of the rare earth to F limits the available connectivity to the silicate tetrahedra, as SiO_3_F tetrahedra are unreported in crystalline structures (Leinenweber et al., 2005; Morrison et al., 2016). Although PO_3_F tetrahedra exist, i.e., Sr(PO_3_F), they have yet to be reported in uranium extended structures, although it is unclear whether this is due to chemical principles or whether the proper conditions for this structure motif have yet to be explored.

Herein we present the synthesis and structural characterization of two examples of uranium oxyfluorides that adopt the phosphuranylite topology, Cs_4.4_K_0.6_[(UO_2_)_6_O_4_F(PO_4_)_4_(UO_2_)] (**3**) and Rb_4.4_K_0.6_[(UO_2_)_6_O_4_F(PO_4_)_4_(UO_2_)] (**4**), and a new sheet anion topology that is observed for both a pure oxide, Cs_11_[(UO_2_)_12_(PO_4_)_3_O_13_] (**1**), and an oxyfluoride, Rb_11_[UO_2_)_12_(PO_4_)_3_O_12_F_2_] (**2**).

## Experimental

### Synthesis

Compounds **1**-**4** were synthesized via molten flux methods using alkali chloride fluxes (Bugaris and zur Loye, 2012; Juillerat et al., 2019a). For all reactions UF_4_ (International Bio-Analytical Industries, powder, ACS grade) was used as the uranium starting material, AlPO_4_ (Alfa Aesar, powder, 99.99%) was used as the phosphate source, and an alkali halide, CsCl (Alfa Aesar, powder, 99.99%), KCl (Mallinckrodt Chemicals, powder, 99.6%), or RbCl (Alfa Aesar, powder, 99.8%), or a mix thereof was used as a flux. ***Caution!*** *Although the uranium precursor used contained depleted uranium, standard safety measures for handling radioactive substances must be followed*. Generally, all solid reactants were loaded into either an alumina or platinum crucible and heated to 875°C in 1.5 h, held at this temperature for 12 h, then cooled at 6°C/h to 550 or 450°C depending on the melting point of the flux. After slow cooling, the furnace was shut off and allowed to rapidly cool to room temperature before sonicating the reaction mixtures in water to remove the flux and isolate the crystalline products by vacuum filtration.

**Figure 1:**
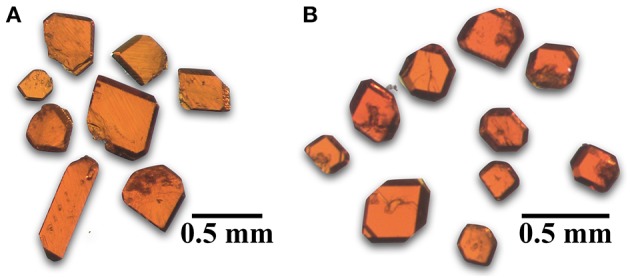
Optical images of orange-red tablet shaped single crystals of **(A)** Cs_11_[(UO_2_)_12_(PO_4_)_3_O_13_] (**1**) and **(B)** Rb_11_[UO_2_)_12_(PO_4_)_3_O_12_F_2_] (**2**).

Cs_11_[(UO_2_)_12_(PO_4_)_3_O_13_] (**1**) was synthesized by loading 1 mmol UF_4_, 0.25 mmol AlPO_4_, and 20 mmol of CsCl in a platinum crucible with a loose-fitting platinum lid and was slow cooled to 550°C. The reaction produced red tablets (Figure 1) in a nearly quantitative yield with no identifiable by-products. Rb_11_[UO_2_)_12_(PO_4_)_3_O_12_F_2_] (**2**) was obtained by loading 0.5 mmol UF_4_, 0.125 mmol AlPO_4_, and 20 mmol of RbCl into a small alumina crucible in a concrete holder with a larger inverted crucible covering it. This mixture was heated as mentioned above and slow cooled to 550°C and produced similar looking orange-red tablets as in **1** in a nearly quantitative yield with no identifiable byproducts. Cs_4.4_K_0.6_[(UO_2_)_6_O_4_F(PO_4_)_4_(UO_2_)] (**3**) and Rb_4.4_K_0.6_[(UO_2_)_6_O_4_F(PO_4_)_4_(UO_2_)] (**4**) were obtained from reactions of 0.5 mmol UF_4_, 0.2 mmol AlPO_4_, and 5 mmol CsCl or RbCl and 5 mmol KCl loaded into an alumina crucible covered with an alumina plate held in place by rubber cement. These were heated and slow cooled to 450°C and produced a yellow crystalline product identified as a mixture of the newly reported F containing phases and either Cs_x_K_4−x_[(UO_2_)_3_(PO_4_)_2_O_2_] orRb_1.4_K_2.6_[(UO_2_)_3_(PO_4_)_2_O_2_] that are visibly indistinguishable. The purity and identity of the products were determined by powder X-ray diffraction (PXRD) using a Bruker D2 Phaser equipped with a LYNXEYE silicon strip detector using a Cu Kα (λ = 1.54056 Å) source.

### Structure

The reported structure solutions were obtained from single crystal X-ray diffraction (SXRD) data collected on a Bruker D8 QUEST diffractometer equipped with an Incoatec IμS 3.0 microfocus radiation source (Mo Kα, λ = 0.71073 Å) and a PHOTON II area detector. The reduction absorption correction was applied to the raw data using SAINT+ and SADABS within the APEX3 software (Bruker, 2015). The SHELXL suite was used within the OLEX2 GUI to solve the structure using SHELXT and refine the solution using SHELXL (Sheldrick, 2015a,b). The TWINROTMAP functionality in PLATON was used to check for missed symmetry elements and twin laws, where **3** and **4** were both refined as two component inversion twins with a significant volume fraction of 0.414(6) for **3** and a minor twin component of 0.084(8) in **4** (Spek, 2009). Full crystallographic information can be found in Table 1.

**Table 1:** Crystallographic details of structures **1**-**4**.

**Formula**	**Cs_11_[(UO_2_)_12_(PO_4_)_3_O_13_]**	**Rb_11_[UO_2_)_12_(PO_4_)_3_O_12_F_2_]**	**Cs_4.4_K_0.6_[(UO_2_)_6_O_4_F(PO_4_)_4_(UO_2_)]**	**Rb_4.4_K_0.6_[(UO_2_)_6_O_4_F(PO_4_)_4_(UO_2_)]**
	**1**	**2**	**3**	**4**
S. G.	*Pnma*	*Pnma*	*Fdd2*	*Fdd2*
a, Å	14.9561(3)	14.1258(3)	25.8529(6)	25.6593(5)
b, Å	17.9663(4)	18.0121(4)	28.9285(6)	27.5792(5)
c, Å	20.8520(4)	20.6241(4)	9.2321(2)	9.2591(2)
V, Å^3^	5603.1(2)	5247.40(19)	6904.6(3)	6552.3(2)
Crystal size (mm^3^)	0.01 × 0.05 × 0.06	0.01 × 0.05 × 0.06	0.01 × 0.04 × 0.06	0.01 × 0.02 × 0.05
Temperature (K)	300	301	300	300
Density (g cm^−3^)	6.158	5.944	5.671	5.549
θ range (°)	2.381–36.355	2.445–36.353	2.446–36.355	2.452–36.343
μ (mm^−1^)	41.774	47.232	37.649	41.374
Collected reflections	234955	190443	177628	168495
Unique reflections	13924	13063	8388	7945
*R*_int−_	0.0485	0.0467	0.0401	0.0171
*h*	*−24 ≤ h ≤ 24*	*−23 ≤ h ≤ 23*	*−43 ≤ h ≤ 43*	*−42 ≤ h ≤ 42*
*k*	*−29 ≤ k ≤ 29*	*−30 ≤ k ≤ 30*	*−48 ≤ k ≤ 48*	*−45 ≤ k ≤ 45*
*l*	*−34 ≤ l ≤ 34*	*−34 ≤ l ≤ 34*	*−15 ≤ l ≤ 15*	*−15 ≤ l ≤ 15*
Δρ*_*max*_ (*e Å^−3^)	3.422	3.155	2.072	1.773
Δρ*_*min*_ (*e Å^−3^)	–5.467	–3.117	–2.471	–2.325
*GoF*	1.111	1.128	1.141	1.081
Extinction coefficient	0.000066(2)	0.000011(2)	–	–
*R_1_(F) for $F_{0}^{2}>$2*σ*($F_{0}^{2}$)*^a^**	0.0262	0.0278	0.0191	0.0179
*R_*w*_($F_{0}^{2}$)*^b^**	0.0473	0.0551	0.0379	0.0402

a *$R_{1}=\Sigma ||F_{0}|-|F_{c}|\;|/\Sigma |F_{0}|$*.

b *$wR_{2}={\left[\Sigma w{\left({F_{0}}^{2}-{F_{c}}^{2}\right)}^{2}/\Sigma w{\left({F_{0}}^{2}\right)}^{2}\right]}^{1/2}$; $P=({F_{0}}^{2}+2{F_{c}}^{2})/3$; $w=1/\left[\sigma^{2}\left({F_{0}}^{2}\right)+{\left(0.038P\right)}^{2}+58.7789P\right]$ for ***1***, $w=1/\left[\sigma^{2}\left({F_{0}}^{2}\right)+{\left(0.0117P\right)}^{2}+51.4853P\right]$ for ***2***, $w=1/\left[\sigma^{2}\left({F_{0}}^{2}\right)+{\left(0.0032P\right)}^{2}+174.9170P\right]$ for ***3***, and $w=1/\left[\sigma^{2}\left({F_{0}}^{2}\right)+{\left(0.0146P\right)}^{2}+96.7826P\right]$ for ***4****.

In all structures the refinement of the U sites is straightforward, while in all structures there is disorder among the alkali cation sites, and in structures **1** and **2** there is disorder in one of the phosphate tetrahedra. Generally, the disorder in the alkali sites was treated by freely refining the sites as Cs or Rb as appropriate, and if less than one, then it was assumed that either the sites is shared by a smaller alkali cation, K^+^ in **3** and **4**, or a disordered site across multiple positions. The presence of significant nearby electron density peaks suggests a disordered site, while the absence of these suggests sharing of the site between Cs/K or Rb/K. Mixed sites, and multiple disordered sites were constrained to occupancies of one using free variables or SUMP commands in cases of more complicated disorder, and the use of ISOR and EADP commands were implemented to constrain thermal parameters. The full details of the structure refinements are located in Supporting Information and the checkoff report is contained in Supplementary Data Sheet 1.

In structures **1** and **2** the P3 site is half occupied because it is disordered across a mirror plane and the two disordered sites are too close to both be fully occupied sites. This is also true for O23, O27, O28 which are coordinated to P3 and O29 coordinated to P1 in **1**, and O28A and F28B in **2**. Labeling all sites within the coordination sphere of the U sites as O in structures **2**-**4**, does not result in charge balance, as there is an excess of negative charge. This could not be resolved by reasonable models of the alkali cation disorder and this observation, along with the identification of F in all three structures by EDS in both powdered and singly crystalline forms, confirms the presence of F. While the fluorine site could be easily located in structure **2** by using bond valence sums (BVS) and knowledge of U coordination chemistry (discussed in structure description), it was not easily identified in **3** and **4**. In **3** and **4** the O3 site was fixed as a half occupied O/F shared site to maintain charge balance in the crystallographic solution, and this arbitrary assignment will be discussed in later sections.

EDS was used to verify the presence of F in **2**-**4** and all other elements present in each single crystal used for structure determination as well as in bulk powder samples of **1** and **2**. Data were collected on a TESCAN Vega-3 SBU equipped with an EDS detector.

### Optical Spectroscopy

UV-vis and fluorescence measurements were performed on bulk powder samples of **1** and **2** using a PerkinElmer Lambda 35 UV-vis scanning spectrophotometer equipped with an integrating sphere and a PerkinElmer LS55 Luminescence spectrometer. The UV-vis diffuse reflectance data were internally converted using the Kulbelka-Munk equation and then normalized (Kubelka and Munk, 1931). Fluorescence excitation spectra were collected at emission wavelengths of 574 and 564 nm for **1** and **2**, respectively, and emission spectra were collected at an excitation wavelength of 437 nm for both **1** and **2**.

### Ion Exchange

Ion exchange experiments were performed on powder and single crystalline samples of Cs_11_[(UO_2_)_12_(PO_4_)_3_O_13_] (**1**) and Rb_11_[UO_2_)_12_(PO_4_)_3_O_12_F_2_] (**2**) where 20 mg of sample was soaked in ~4 mL of concentrated salt solution in a drying oven set to 90°C for 3 days. The Rb analog, **2**, was soaked in 11 m CsCl solutions while the Cs analog, **1**, was soaked in 7 m RbCl or 4 m KCl solutions. Products were examined by EDS and PXRD as described above.

### First Principles Calculations

We used first-principles calculations using the density functional theory (DFT) code VASP (Vienna Ab-initio Simulation Package) (Kresse and Furthmuller, 1996a,b) employing the projector augmented wave (PAW) method (Blöchl, 1994; Kresse and Joubert, 1999) and generalized gradient approximation of Perdew, Burke and Ernzerhof (PBE) (Perdew et al., 1996) to model the systems. These were spin-polarized calculations, using a plane wave basis set with an energy cut-off of 520 eV to expand the electronic wave functions, and 10^−6^ eV energy convergence criteria. A 2 × 2 × 2 **k**-point mesh was used for sampling the Brillouin zone. The ground state geometries at 0 K were obtained by relaxing the cell volume, atomic positions, and cell symmetry until the maximum forces on each atom were < 0.01 eV/Å. To better represent the correlated nature of the U *f*-electrons, we employed the DFT+*U* method (Anismov et al., 1993; Liechtenstein et al., 1995), with a *U*_eff_ for the U atoms of 4.0 eV (*U*_eff_ = *U* – *J*, with *U* = 4.0 eV, and *J* = 0.0 eV). The *U*_eff_ value was chosen to be close to that obtained from related experimental results for UO_2_ (Schoenes, 1987; Kotani and Takao, 1992). The valence electron configurations were [U] 6s^2^6p^6^5f^3^6d^1^7s^2^, [Cs] 5s^2^5p^6^6s^1^, [P] 3s^2^3p^3^, [O] 2s^2^2p^4^, and [F] 2s^2^2p^5^, respectively.

In an effort to understand why Rb forms an oxyfluoride while Cs only an oxide, we also considered the two opposite cases, Rb_11_(UO_2_)_12_(PO_4_)_3_O_13_ and Cs_11_(UO_2_)_12_(PO_4_)_3_O_13_F_2_. Because the oxide and oxyfluoride compounds have different composition, we cannot directly compare their calculated total energies, i.e., thermodynamic stability. Therefore, we need to investigate their relative stability by analyzing the reaction enthalpies, Δ_r_*H*, considering the two reactions:

(1) $$\begin{array}{r} \;12{\text{UF}}_{4}+3{\text{AlPO}}_{4}+11\text{RbCl}+37/2{\text{O}}_{2}\to \\ {\text{Rb}}_{11}{\left({\text{UO}}_{2}\right)}_{12}{\left({\text{PO}}_{4}\right)}_{3}{\text{O}}_{12}{\text{F}}_{2}+3{\text{AlF}}_{3}+37/2{\text{F}}_{2}+11/2{\text{Cl}}_{2} \end{array}$$

(2) $$\begin{array}{r} \;12{\text{UF}}_{4}+3{\text{AlPO}}_{4}+11\text{CsCl}+37/2{\text{O}}_{2}\to \\ {\text{Cs}}_{11}{\left({\text{UO}}_{2}\right)}_{12}{\left({\text{PO}}_{4}\right)}_{3}{\text{O}}_{13}+3{\text{AlF}}_{3}+39/2{\text{F}}_{2}+11/2{\text{Cl}}_{2} \end{array}$$

The Δ_r_*H*, values were calculated using:

(3) $$\Delta_{r}H=\;\sum_{i=products}c_{i}\Delta_{f}H\left(i\right)-\;\sum_{j=reactants}c_{j}\Delta_{f}H\left(j\right),$$

where Δ_*f*_*H* are the formation energies per formula unit of the products, *i*, and reactants, *j*, and the sum is over all products and reactants. *c*_*i*_ and *c*_*j*_ are the stoichiometric coefficients of the products and reactants, respectively. For each of the reactants and products we calculated their Δ_*f*_*H* using the same VASP calculations input parameters listed above.

DFT gives only the reaction enthalpy at 0 K, and to include the temperature effect on the reactions, we calculate the finite temperature quasi-Gibbs formation energies, gf, using the equation:

(4) $$\Delta_{f}g=\Delta_{f}H-TS_{conf}$$

*S*_conf_ is the configurational entropy, defined as:

(5) $$S_{\text{conf}}=k_{b}\sum_{i}x_{i}\ln\left(x_{i}\right)$$

where *k*_b_ is the Boltzmann constant, *x*_*i*_ is the mole fraction of the constituent *i*, and the sum is over each constituent *i* in the compound. Note that in our calculations we do not consider the vibrational contribution to the entropy because due to their very large size, calculating this term for the title compounds is outside the current capabilities of DFT, and hence the term quasi-Gibbs energy. In the case of the gases O_2_, F_2_, and Cl_2_, we use tabulated values for the standard entropies (Chase, 1998).

## Discussion

### Synthesis

There have been numerous reported uranium phosphate containing structures prepared by similar synthetic methods using UF_4_, AlPO_4_, and alkali chloride fluxes, predominantly loaded into alumina crucibles and heated at a temperature of 875°C (Juillerat et al., 2018a,b; Juillerat and zur Loye, 2019; Juillerat et al., 2019b). Cs_11_[(UO_2_)_12_(PO_4_)_3_O_13_] (**1**) and Rb_11_[UO_2_)_12_(PO_4_)_3_O_12_F_2_] (**2**) were first discovered as the minor product, previously unidentified red tablets, in the synthesis of Cs_6_[(UO_2_)_7_O_4_(PO_4_)_4_] and Rb_6_[(UO_2_)_7_O_4_(PO_4_)_4_], respectively (Juillerat et al., 2018b). The optimization of the Cs containing analog lead to the use of platinum crucibles, and was successful, but when using analogous synthetic techniques for the Rb analog, only simple rubidium oxides, such as Rb_2_U_2_O_7_, were obtained in platinum crucibles. The use of alumina crucibles for the synthesis of Rb_11_[UO_2_)_12_(PO_4_)_3_O_12_F_2_] (**2**) proved necessary, although the reason is not well-understood. Structures **3** and **4**, related to the phosphuranylite topology, were discovered when trying to optimize synthetic conditions for Cs_1.4_K_2.6_[(UO_2_)_3_O_2_(PO_4_)_2_], Cs_1.7_K_4.3_[(UO_2_)_5_O_5_(PO_4_)_2_], Rb_1.4_K_2.6_[(UO_2_)_3_O_2_(PO_4_)_2_] and Rb_1.6_K_4.4_[(UO_2_)_5_O_5_(PO_4_)_2_]. The synthesis of these phases is described and discussed in a recent publication which concluded that higher flux to reactant ratios (40 mmol flux to 0.5 mmol UF_4_) favored the formation of the phases Cs_1.4_K_2.6_[(UO_2_)_3_O_2_(PO_4_)_2_] and Rb_1.4_K_2.6_[(UO_2_)_3_O_2_(PO_4_)_2_], while 20 mmol of flux and 0.5 mmol UF_4_ lead to synthesis of Cs_1.4_K_2.6_[(UO_2_)_3_O_2_(PO_4_)_2_] and Cs_1.7_K_4.3_[(UO_2_)_5_O_5_(PO_4_)_2_] or Rb_1.4_K_2.6_[(UO_2_)_3_O_2_(PO_4_)_2_] and Rb_1.6_K_4.4_[(UO_2_)_5_O_5_(PO_4_)_2_], which could not be successfully separated (Juillerat et al., 2018a). The title phases, Cs_4.4_K_0.6_[(UO_2_)_6_O_4_F(PO_4_)_4_(UO_2_)] (**3**) and Rb_4.4_K_0.6_[(UO_2_)_6_O_4_F(PO_4_)_4_(UO_2_)] (**4**), were obtained simultaneously with Cs_1.4_K_2.6_[(UO_2_)_3_O_2_(PO_4_)_2_] and Rb_1.4_K_2.6_[(UO_2_)_3_O_2_(PO_4_)_2_] by further reducing the flux to reactants ratio to 10 mmol and 5 mmol of flux with 0.5 mmol UF_4_; unfortunately, the title compounds could not be separated manually from Cs_1.4_K_2.6_[(UO_2_)_3_O_2_(PO_4_)_2_] and Rb_1.4_K_2.6_[(UO_2_)_3_O_2_(PO_4_)_2_], as all crystallize as yellow plates.

Cs_4.4_K_0.6_[(UO_2_)_6_O_4_F(PO_4_)_4_(UO_2_)] (**3**) and Rb_4.4_K_0.6_[(UO_2_)_6_O_4_F(PO_4_)_4_(UO_2_)] (**4**) contain relatively small amounts of potassium, and attempts were made to synthesize potassium-free Cs and Rb analogs of this uranyl oxyfluoride based on the phosphuranylite sheet topology; however, none were successful as pure CsCl or RbCl fluxes lead to the synthesis of **1**, **2**, Cs_6_[(UO_2_)_7_O_4_(PO_4_)_4_], Rb_6_[(UO_2_)_7_O_4_(PO_4_)_4_], Cs_3_[Al_2_O(PO_4_)_3_][(UO_2_)_3_O_2_], Rb_3_[Al_2_O(PO_4_)_3_][(UO_2_)_3_O_2_], or a mixture of these products.

### Structure

**Figure 2:**
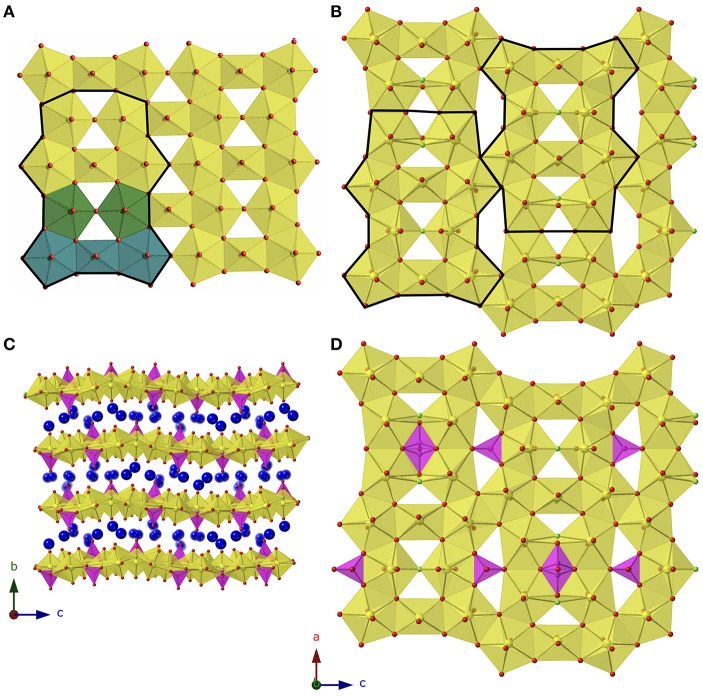
**(A)** β-U_3_O_8_ topology with building unit outlined in black and U_3_O_16_ and U_2_O_14_ units shown in blue and green, respectively. **(B)** Topology of **1** and **2** with the building unit boldened, and the phosphate tetrahedra omitted. **(C)** View of **1** in the *a* direction. **(D)** The sheets found in **2** with phosphate tetrahedra included. Uranium, phosphorus, oxygen, and alkali cations are shown in yellow, magenta, red, and blue, respectively.

Cs_11_[(UO_2_)_12_(PO_4_)_3_O_13_] (**1**) and Rb_11_[UO_2_)_12_(PO_4_)_3_O_12_F_2_] (**2**) are two dimensional sheet structures (Figure 2C) and crystallize in the orthorhombic space group *Pnma* with lattice parameters *a* = 14.9561(3), *b* = 17.9663(4), *c* = 20.8520(4), and *a* = 14.1258(3), *b* = 18.0121(4), *c* = 20.6241(4), respectively. To our knowledge, the sheet topology present in both structures is unreported in the literature and can be deconstructed into units of the β-U_3_O_8_ topology as shown in Figure 2A. The β-U_3_O_8_ topology can be deconstructed into U_3_O_16_ and U_2_O_14_ units, where the U_3_O_16_ unit is a square bipyramid, edge-sharing with two pentagonal bipyramids on either side, and the U_2_O_14_ unit is two corner-sharing pentagonal bipyramids. These two units alternate to form the β-U_3_O_8_ topology as shown in Figure 2A. Structure **1** is also built-up of alternating U_3_O_16_ and U_2_O_14_ units and **2** contains the U_3_O_16_, U_2_O_14_, and U_2_O_13_F units where the pentagonal bipyramids corner share through the F; however, every third U_3_O_16_ unit is missing in both structures (Figure 2B).

**Figure 3:**
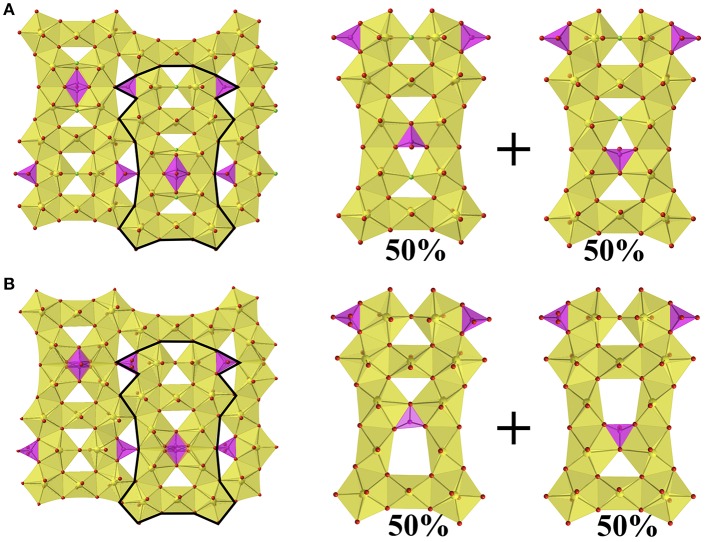
**(A)** Sheets found in Rb_11_[UO_2_)_12_(PO_4_)_3_O_12_F_2_] (**2**) showing the two possible orientations of the phosphate tetrahedron, where F in presence in the absence of the phosphate tetrahedron. **(B)** The sheets found in Cs_11_[(UO_2_)_12_(PO_4_)_3_O_13_] (**1**) showing the two possible orientations of the phosphate tetrahedron.

Between the group of four pentagonal bipyramids (two edge sharing U_2_O_14_ or U_2_O_13_F units) is a disordered phosphate tetrahedra with two possible orientations as shown in Figure 2D. Additional phosphate tetrahedra edge share with the U_2_O_14_/U_2_O_13_F units that are located between two U_3_O_16_ units and corner share to the adjacent group of four pentagonal bipyramids. The disorder in the phosphate tetrahedron, P3 in both structures **1** and **2**, is slightly different between the two structures as shown in Figure 3. In Figure 3A, depicting Rb_11_[UO_2_)_12_(PO_4_)_3_O_12_F_2_] (**2**), the phosphate tetrahedron is half occupied and accompanied by a split oxygen/fluorine site whose occupancies sum to 1. Therefore, either the phosphate tetrahedron points up or down (with respect to the plane of the sheet), where the oxygen site corresponds to the orientation of the phosphate tetrahedron, and the fluorine site corresponds to the absence of the phosphate tetrahedron. This is supported by the P3-O28A and P3-F28B bond distances which are 1.555(8) and 2.170(8) Å, respectively where the P-F bond is much too long for the tetrahedral coordination environment of P^5+^. Tables of bond valences and bond distances for U and P for all structures are collected in Tables S1–S4.

The disorder in the P3 tetrahedron in the Cs analog, structure **1**, is similar by virtue of the phosphate tetrahedron pointing up or down within the plane of the sheet; however, the electron density near the O28 site freely refines to a half occupied oxygen site (rather than unity as in **2**), and therefore in the absence of the phosphate tetrahedron there are two square bipyramids (Figure 3B) as opposed to two corner-sharing pentagonal bipyramids as in **2**. The bond valence sums of U6 and U7, the corner-sharing pentagonal bipyramids in **2** and the two square bipyramids created by the absence of P3 in **1**, are slightly lower at values of 5.749 and 5.767, respectively, for **1**, as compared to 5.844 and 5.833, respectively for **2**; however, all values are within the accepted range for U^6+^ (~5.6–6.1) (Burns et al., 1997). Tables of bond valences and bond distances for U and P for all structures are located in Tables S1–S4.

While EDS identifies the presence (or absence) of fluorine in structures **1** and **2**, it does not identify the positions of the fluorine sites within structure **2**. In order to locate the F sites in **2** we calculated BVS for all possible F sites, which includes all oxygen sites coordinated to the uranium sites except for the axial uranyl oxygens, as F on a uranyl oxygen site would be extremely unexpected given the bond order of the ~1.8 Å U-O “yl” bond (3) and multiple bonds are not possible for F. All of the uranyl U-O bond lengths in **2** are between 1.802(4) and 1.830(6) Å and show no significant deviation from the expected ~1.8 Å bond length. Bond valence sums of the remaining O atoms are between 1.82 and 2.30 using *r*_*o*_ = 2.051 and *B* = 0.519 for U-O (Burns et al., 1997), *r*_*o*_ = 1.617 and *B* = 0.370 for P-O (Brown and Altermatt, 1985), and *r*_*o*_ = 2.081 and *B* = 0.515 for Rb-O (Brown and Altermatt, 1985), except for “O5” and “O28B” which have low values of 1.42 and 1.28, and which are significantly lower than the expected value of 2. If *r*_*o*_ = 1.98 and *B* = 0.40 for U-F bonds (Zachariasen, 1978), these bond valence sums come out to 1.02 and 0.94, respectively, and match well with the expected value of 1 for F; therefore, these sites have been identified as F5 and F28B and are necessary for achieving charge balance in the structure. For comparison, the bond valence sums for O5 and O28 sites in structure **1**, that contains no fluorine, are 1.86 and 1.90, respectively, and therefore these results also support our decision to assign the fluorine sites in **2** as F5 and F28B.

Cs_4.4_K_0.6_[(UO_2_)_6_O_4_F(PO_4_)_4_(UO_2_)] (**3**) and Rb_4.4_K_0.6_[(UO_2_)_6_O_4_F(PO_4_)_4_(UO_2_)] (**4**) also contain small amounts of fluorine as identified by EDS and by the need for charge balance in the structures. Similar methods as described above were used to identify the fluorine sites(s), again excluding uranyl oxygen sites as possibilities; however, the results are less definitive than the bond valence sums of **2**. In **3** all O bond valence sums are between 1.92 and 2.22, none of which signal good candidates for fluorine doping, and similarly in **4**, the O BVS are between 1.78 and 2.25. This suggests that there is no preferred site for F and for this reason we have arbitrarily set the occupancy of O3 to be half occupied by both F and O, as this site has the lowest BVS in both structures **3** and **4**, and the rarity of PO_3_F tetrahedra leaves O3 and O6 as the most plausible options. [(UO_2_)_5_(HPO_4_)_3_(PO_4_)F_4_](H_9_C_10_N_2_)_3_ synthesized hydrothermally using ${\text{PF}}_{6}^{-}$ as the F^−^ source also contains phosphuranylite related layers and fluorine is present on sites similar to the ones found in **3** and **4** (Deifel et al., 2008).

**Figure 4:**
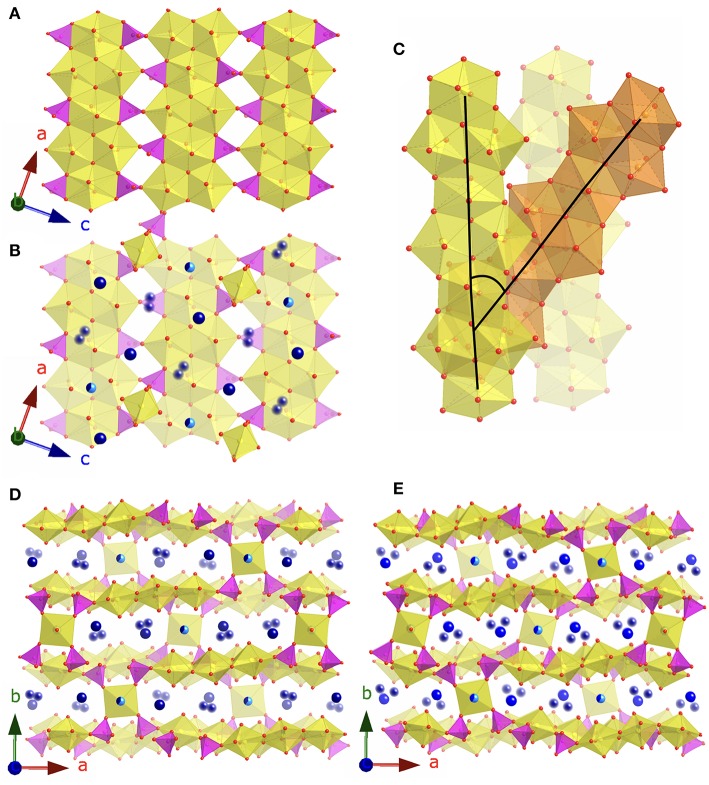
**(A)** The phosphuranylite layers found in **3** and **4. (B)** The phosphuranylite layers plus the square bipyramid uranyl sites and alkali cations. **(C)** Depiction of the 37.8° torsion angle between chains in adjacent layers. **(D)** View of **3** in the *c* direction and **(E)** view of **4** in the *c* direction. Uranium, phosphate, and oxygen are shown in yellow, magenta, and red, respectively. Alkali cations are shown in blues, where blurred spheres represent partially occupied disordered sites, and the half light/dark blue spheres represent the Cs/K and Rb/K shared sites.

The Cs_4.4_K_0.6_[(UO_2_)_6_O_4_F(PO_4_)_4_(UO_2_)] (**3**) and Rb_4.4_K_0.6_[(UO_2_)_6_O_4_F(PO_4_)_4_(UO_2_)] (**4**) structures are similar to the phosphuranylite mineral, KCa(H_3_O)_3_[(UO_2_)_6_O_4_(PO_4_)_4_(UO_2_)(H_2_O)_8_] (Demartin et al., 1991), as they contain the same phosphuranylite-type layers constructed of chains of alternating UO_8_ and U_2_O_12_ units connected to adjacent chains through corner- and edge- sharing phosphate tetrahedra (Figure 4A). There is also an additional uranium site, square bipyramid, corner sharing with four phosphate tetrahedra to link adjacent sheets, as there is in phosphuranylite (Figure 4B). The alkali sites fill the voids between the uranyl phosphate layers (Figures 4D,E). Several recent papers have further characterized phosphuranylite type layers by the direction in which the phosphate tetrahedra point (up or down orthogonal to the plane of the sheet) and there are seven known isomers (Locock and Burns, 2003; Juillerat et al., 2018a; Juillerat and zur Loye, 2019). Both structures **3** and **4** are the uudduuO isomer, observed in the mineral phurcalite (Atencio et al., 1991), while phosphuranylite is the uudduuS isomer. In addition to the presence of fluorine in the uranyl phosphate sheets, the stacking of the uranyl phosphate sheets in **3** and **4** is also different from those observed in other phosphuranylite based structures. In phosphuranylite and recently reported phosphuranylite type structures, the chains of UO_8_ and U_2_O_12_ units in adjacent sheets run parallel to each other, while in **3** and **4** there are two orientations of these chains that alternate between layers, where the torsion angle between chains in two adjacent layers is 37.8°. This is illustrated in Figure 4C where parallel layers are shown in yellow, and the layer between these is shown in orange.

### First-Principles Calculations

As was mentioned previously, Cs prefers to form the oxide structure, while Rb prefers the oxyfluoride structure, and to understand the cause of this different behavior, we studied their reaction enthalpies using DFT. Shown in Table 2 are the calculated Δ_r_*H* values which indicate reactions (1) and (2) are thermodynamically unfavorable, i.e., their Δ_r_*H* values are positive. Also, the [A, O_12_F_2_] compounds have more negative Δ_r_*H* compared to the respective [A, O_13_] compound, indicating that forming the [A, O_12_F_2_] is preferred over the [A, O_13_]. Experimental results confirm that this is the case for the Rb containing compound, it disagrees for that containing Cs. This discrepancy between experiment and calculations may come from the fact that the calculations are performed at 0 K, and thus, finite temperature enthalpy values could provide a different conclusion.

**Table 2:** Reaction enthalpies (Δ_r_*H*) and difference in reaction enthalpies (ΔΔ_r_*H*), in kJ/mol/atom, of the A_11_(UO_2_)_12_(PO_4_)_3_O_12_F_2_ ([A, O_12_F_2_]) and A_11_(UO_2_)_12_(PO_4_)_3_O_13_ ([A, O_13_]) compounds (A = Rb, Cs).

**A_11_**	**[A, O_12_F_2_]**	**[A, O_13_]**	**ΔΔ_r_*H* ([A, O_13_] – [A, O_12_F_2_])**
Rb	47.02	53.90	6.88
Cs	47.14	51.23	4.09

**Figure 5:**
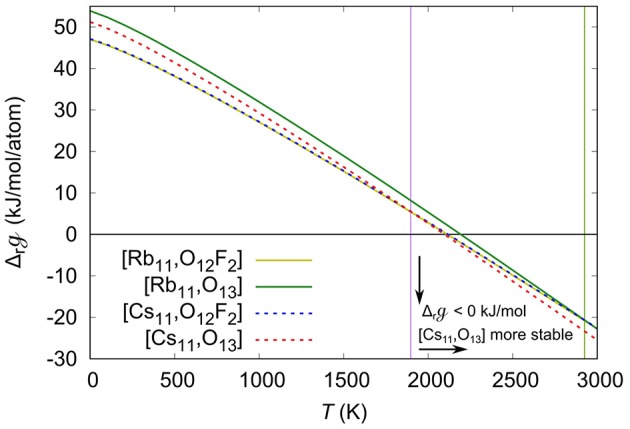
Quasi-Gibbs reaction energies ($\Delta_{r}g$), in kJ/mol/atom, as a function of the temperature (T), in K. The Δ_r_*G* of the Rb_11_(UO_2_)_12_(PO_4_)_3_O_12_F_2_ ([Rb,O_12_F_2_]), Rb_11_(UO_2_)_12_(PO_4_)_3_O_13_ ([Rb,O_13_]) Cs_11_(UO_2_)_12_(PO_4_)_3_O_12_F_2_ ([Cs,O_12_F_2_]), and Cs_11_(UO_2_)_12_(PO_4_)_3_O_13_ ([Cs,O_13_]) are shown in yellow, green, blue and red lines, respectively. The purple and green vertical lines respectively show the temperature at which the CsO_13_ and RbO_13_ become more stable than the CsO_12_F_2_ and RbO_12_F_2_.

To consider the temperature effect, we calculated the gf using Equation (4), and substituted them in Equation (3) to obtain the quasi-Gibbs reaction energies, gr. Shown in Figure 5 is the calculated gr as a function of the temperature. With increasing temperature the gr becomes more negative, eventually becoming < 0 at *T* > 2,200 K, implying that above that temperature the reactions are thermodynamically favorable. It is also important to note that the gr of the [Cs, O_13_] compound becomes more negative than the gr of the [Cs, O_12_F_2_] compound for *T* > 1,900 K, the temperature at which a phase change occurs. Moreover, above the temperature at which the gr becomes negative, the [Cs, O_13_] gr is more negative compared to the [Cs, O_12_F_2_] gr, indicating that above 2,200 K the formation of [Cs, O_13_] is thermodynamically preferred over the formation of [Cs, O_12_F_2_]. The results suggest that the formation of the oxide is driven by the entropy, and that at high enough temperatures, above 2,925 K from our calculations, the [Rb, O_13_] can also be formed over the [Rb, O_12_F_2_]. Also, note that the difference between the phase change temperatures of the Cs and Rb compounds is 1,025 K, which is big enough so that a phase change is observed in one case but not the other. However, the calculated reaction temperature, ~2,200 K is much higher than the experimental one, 1,148 K. The difference arises from the absence of the other entropic contributions in our calculations, except for the gases. Because of the large size of the systems we are not able to calculate the phonon spectra, whereas introducing the vibrational contribution might lower the calculated reaction temperature to better match the experimental results.

### Ion Exchange

The ion exchange products of **1** soaked in RbCl and KCl and **2** soaked in CsCl show small changes in the PXRD patterns shown in Figures S1, S2, indicating the layered structures are maintained throughout the ion exchange process. EDS was used to qualitatively analyze the alkali contents of each sample before and after ion exchange. The results showed that after 3 days of soaking in concentrated salt solution approximately half of the alkali species are exchanged. In the exchange of Cs in **1** for Rb and K, the approximate contents of the post ion-exchange products are 5.3 Cs, 5.7 Rb, and 5.4 Cs, 5.6 K, respectively. While in the exchange of Rb for Cs in **2**, results in 6.3 Rb, 4.7 Cs.

### Optical Properties

The UV-vis absorption spectra (Figure S3) and fluorescence emission spectra (Figure S4) of Cs_11_[(UO_2_)_12_(PO_4_)_3_O_13_] (**1**) and Rb_11_[UO_2_)_12_(PO_4_)_3_O_12_F_2_] (**2**) are typical of U^6+^ species in uranium oxide extended structures. **1** and **2** absorb broadly from 200 to 575 nm and can be classified as semiconductors. A careful look at the DFT calculated density of states (Figure S5) showed that these compounds are actually Mott insulators. The ligand to metal charge transfer transitions are at 377 and 379 nm, respectively and the transitions from the ${\text{UO}}_{2}^{2+}$ core are at 473 and 463 nm, respectively. The maximum fluorescence emission occurs at an excitation wavelength of 437 nm where the emission peak is centered on 574 and 564 nm, respectively for **1** and **2**.

## Conclusions

Three new uranyl phosphate oxyfluorides, and one uranyl phosphate, have been synthesized by molten flux methods using alumina crucible reaction vessels and alkali chloride fluxes. Their structures were determined by SXRD and the presence of fluorine was confirmed by qualitative EDS. The location of the fluorine sites was deduced using bond valence sums, although they were inconclusive for determining the location of F in **3** and **4**. Structures **1** and **2** were further characterized by PXRD, DFT calculations, ion-exchange experiments, and optical spectroscopy. The DFT calculations support the observation of the Rb, oxyfluoride structures (**2**) in contrast to the pure Cs, oxide structure of (**1**), indicating that the formation of pure oxide structure may be driven by entropy, and it might be obtained for both Rb and Cs, given high enough temperatures. The temperature difference in the temperatures at which the pure oxide structures can be obtained is 1,025 K between the Rb and Cs, which hints at the reason why we see an oxyfluoride in the Rb containing (**2**) and the pure oxide in the Cs (**1**) containing phases. Structures **1** and **2** are capable of alkali ion exchange, where approximately half of the alkali cations in the parent structure can be replaced by a target alkali species in concentrated salt solutions, although the Cs containing structure, **1**, undergoes more extensive ion exchange than the Rb analog, **2**, perhaps due to the larger interlayer spacing in **1**. Alkali chloride fluxes continue to be a viable synthetic technique for crystallizing new uranium phosphate structures containing new structure types and further exploration should continue, in addition to expanding to alkaline fluxes in order to incorporate divalent cations and hopefully obtain new novel structure types.

## Data Availability

CCDC deposition numbers 1898442–1898445 contain the supplementary crystallographic data for this paper. These data can be obtained free of charge via www.ccdc.cam.ac.uk/data_request/cif, or by emailing data_request@ccdc.cam.ac.uk, or by contacting The Cambridge Crystallographic Data Center, 12 Union Road, Cambridge CB21EZ, UK; fax: +44 1223 336033.

## Author Contributions

CJ and H-CzL were responsible for the synthesis and structural characterization of the four new compounds and most of the written manuscript. VK and TB contributed the DFT calculations, methods, and discussion.

### Conflict of Interest Statement

The authors declare that the research was conducted in the absence of any commercial or financial relationships that could be construed as a potential conflict of interest.

## Supplementary Material

An extended discussion of the crystallographic refinements of **1** and **2**, additional details on the DFT calculations, bond valence sums and selected bond distances, powder X-ray diffraction data, optical spectra of **1** and **2**, and DFT calculated density of states can be found in the Supporting Information.
