# Supplementary material for: Observation of the Same New Sheet Topology in Both the Layered Uranyl Oxide-Phosphate Cs11[(UO2)12(PO4)3O13] and the Layered Uranyl Oxyfluoride-Phosphate Rb11[(UO2)12(PO4)3O12F2] Prepared by Flux Crystal Growth
Source: Front Chem. 2019 Aug 21;7:583. doi: 10.3389/fchem.2019.00583 (PMC6712084; doi:10.3389/fchem.2019.00583)
Supplement: Supplementary file 1 [file Table_1.DOCX]

**Supporting Information**

**Observation of the Same New Sheet Topology in Both the Layered Uranyl Oxide-Phosphate Cs_11_[(UO_2_)_12_(PO_4_)_3_O_13_] and the Layered Uranyl Oxyfluoride-Phosphate Rb_11_[UO_2_)_12_(PO_4_)_3_O_12_F_2_] Prepared by Flux Crystal Growth**

Christian A. Juillerat,^†§^ Vancho Kocevski,^‡§^ Theodore M. Besmann, ^‡§^ Hans-Conrad zur Loye,^*† §^

^†^Department of Chemistry and Biochemistry, University of South Carolina, Columbia, SC, 29208, United States

^‡^Nuclear Engineering Program, University of South Carolina, Columbia, SC, 29208, United States

^§^Center for Hierarchical Wasteform Materials (CHWM), University of South Carolina, Columbia, SC, 29208, United States

**Additional details on the crystallographic refinement:**

In structures **1** and **2** there is severe disorder in the Cs/Rb alkali cations that reside between the uranium phosphate sheets. In **1**, Cs1 is split across three sites, Cs1A, Cs1B, and Cs1C with occupancies of 0.528(10), 0.274(14), 0.196(17), respectively, where the sum of the three is constrained to 1 using a SUMP command. Cs2A/2B and Cs4A/4B are constrained to an occupancy of 1 using a free variable resulting in occupancies of 0.63(2)/0.37(2), and 0.50(4)/0.50(4), respectively. A SUMP command was necessary for constraining sites Cs7A and Cs7B, as Cs7B is projected through a mirror plane, and therefore the sum of Cs7A and two Cs7B were constrained to one. Additionally, an ISOR restraint was used on Cs1B and Cs1C to enforce reasonable thermal displacement parameters. The Rb cations in **2** were handled in a similar manner, where Rb1A/1B, Rb3A/3B, Rb4A/4B were constrained using free variables that resulted in occupancies of 0.913(3)/0.087(3), 0.83(3)/0.17(3), and 0.921(4)/0.079(4), respectively, and sites Rb7A, Rb7B, and Rb7C were constrained using a SUMP command resulting in occupancies of 0.043(3), 0.187(13), and 0.0969(19), respectively.

Similar methods were used in the refinement of **3** and **4**; however, these structures contain two different alkali species. In both structures the A1 site is shared by both Cs (or Rb) and K. This was determined by freely refining the sites as Cs (or Rb) and the absence of additional q-peaks and an occupancy level significantly less than one suggests the site is best modeled as a mixture of Cs/K or Rb/K. In **3** the Cs1/K1 occupancies are 0.389(5)/0.611(5) and in **4** the Rb1/K1 occupancies are similar at 0.413(11)/0.587(11). In both structures there is disorder on the A3 site, split across two sites, A3A and A3B, with occupancies of 0.837(2) and 0.163(2) for Cs3A/Cs3B in **3** and 0.700(2) and 0.300(2) for Rb3A/Rb3B in **4**.

**Additional details on the DFT calculations:**

We used the experimentally generated CIF files for the Rb_11_(UO_2_)_12_(PO_4_)_3_O_12_F_2_ and Cs_11_(UO_2_)_12_(PO_4_)_3_O_13_ compounds as starting structures in our DFT calculations. As mentioned previously, the alkali atom sites were freely refined, yielding a multiple alkali atom sites with different occupancies. To model the alkali atom partial occupancies, we would need to generate a very large supercell, which would be prohibitively costly to run DFT calculations. Therefore, when generating the structures for the DFT calculations, we took the weighted average of the neighboring alkali atoms as the alkali atom position. Also, we made sure that the generated structures have the same symmetry as the experimentally synthesized compounds.

**Table S1:** Bond valence sums and bond distances for Cs_11_[(UO_2_)_12_(PO_4_)_3_O_13_] (**1**).

| Interaction | Distance | Interaction | Distance | Interaction | Distance |
| --- | --- | --- | --- | --- | --- |
| U1 – O1 | 1.833(5) | U4-O4 | 2.221(3) | U6-O12 | 2.180(3) |
| U1 – O2 | 1.822(5) | U4-O9 | 2.222(3) | U6-O19 | 2.141(3) |
| U1 – O3 x 2 | 2.541(3) | U4-O14 | 2.253(3) | U6-O20 | 1.825(3) |
| U1 – O4 x 2 | 2.296(3) | U4-O15 | 1.840(4) | U6-O21 | 1.825(4) |
| U1 – O5 | 2.103(5) | U4-O16 | 1.823(4) | U6-O22 | 2.482(2) |
| **BVS U1** | **6.007** | U4-O19 | 2.275(3) | U6-O23 x 0.5 | 2.276(5) |
| U2-O6 | 1.820(5) | **BVS U4** | **5.820** | U6-O23 x 0.5 | 2.697(5) |
| U2-O7 | 1.816(6) | U5-O3 | 2.380(3) | U6-O28 x 0.5 | 2.738(6) |
| U2-O8 x 2 | 2.579(3) | U5-O4 | 2.263(3) | **BVS U6** | **5.749** |
| U2-O9 x 2 | 2.281(3) | U5-O12 | 2.319(3) | U7-O13 | 2.191(3) |
| U2-F5 | 2.127(5) | U5-O13 | 2.327(3) | U7-O14 | 2.143(4) |
| **BVS U2** | **5.873** | U5-O17 | 1.817(3) | U7-O24 | 1.819(4) |
| U3-O8 | 2.382(3) | U5-O18 | 1.821(3) | U7-O25 | 1.816(4) |
| U3-O9 | 2.281(3) | U5-O19 | 2.461(3) | U7-O26 | 2.462(2) |
| U3-O10 | 1.811(5) | **BVS U5** | **5.962** | U7-O27 x 0.5 | 2.294(6) |
| U3-O11 | 1.805(4) | P1-O3 x 2 | 1.527(4) | U7-O27 x 0.5 | 2.756(6) |
| U3-O12 | 2.335(3) | P1-O26 | 1.556(5) | U7-O28 x 0.5 | 2.730(6) |
| U3-O13 | 2.310(3) | P1-O29 | 1.512(8) | **BVS U7** | **5.767** |
| U3-O14 | 2.488(4) | **BVS P1** | **5.058** | P3-O23 | 1.559(6) |
| **BVS U3** | **5.981** | P2-O8 x 2 | 1.536(4) | P3-O27 | 1.544(6) |
|  |  | P2-O22 | 1.565(4) | P3-O28 | 1.559(6) |
|  |  | P2-O30 | 1.481(6) | P3-O31 | 1.496(6) |
|  |  | **BVS P2** | **5.086** | **BVS P3** | 4.944 |

**Table S2:** Bond valence sums and bond distances for Rb_11_[UO_2_)_12_(PO_4_)_3_O_12_F_2_] (**2**).

| Interaction | Distance | Interaction | Distance | Interaction | Distance |
| --- | --- | --- | --- | --- | --- |
| U1 – O1 | 1.830(6) | U4-O4 | 2.255(3) | U6-O12 | 2.229(3) |
| U1 – O2 | 1.812(6) | U4-O9 | 2.244(3) | U6-O19 | 2.156(3) |
| U1 – O3 x 2 | 2.451(4) | U4-O14 | 2.225(3) | U6-O20 | 1.812(4) |
| U1 – O4 x 2 | 2.222(3) | U4-O15 | 1.837(4) | U6-O21 | 1.812(4) |
| U1 – F5 | 2.245(5) | U4-O16 | 1.821(4) | U6-O22 | 2.557(3) |
| **BVS U1** | **6.095** | U4-O19 | 2.240(3) | U6-O23 | 2.446(2) |
| U2-O6 | 1.822(6) | U4-F28B x 0.5 | 2.931(8) | U6-O28A x 0.5 | 2.569(8) |
| U2-O7 | 1.804(6) | **BVS U4** | **5.935** | U6-F28B x 0.5 | 2.554(7) |
| U2-O8 x 2 | 2.491(4) | U5-O3 | 2.371(4) | **BVS U6** | **5.844** |
| U2-O9 x 2 | 2.228(4) | U5-O4 | 2.327(3) | U7-O13 | 2.244(3) |
| U2-F5 | 2.276(5) | U5-O12 | 2.333(3) | U7-O14 | 2.165(4) |
| **BVS U2** | **5.939** | U5-O13 | 2.289(3) | U7-O24 | 1.802(4) |
| U3-O8 | 2.393(4) | U5-O17 | 1.812(4) | U7-O25 | 1.810(4) |
| U3-O9 | 2.341(4) | U5-O18 | 1.817(4) | U7-O26 | 2.536(3) |
| U3-O10 | 1.821(4) | U5-O19 | 2.329(3) | U7-O27 | 2.460(3) |
| U3-O11 | 1.812(4) | **BVS U5** | **6.080** | U7-O28A x 0.5 | 2.614(7) |
| U3-O12 | 2.301(3) | P1-O3 x 2 | 1.521(4) | U7-F28B x 0.5 | 2.558(7) |
| U3-O13 | 2.320(4) | P1-O26 | 1.521(6) | **BVS U7** | **5.833** |
| U3-O14 | 2.343(4) | P1-O29 | 1.492(10) | P3-O23 | 1.534(5) |
| **BVS U3** | **6.015** | **BVS P1** | **5.256** | P3-O27 | 1.525(6) |
|  |  | P2-O8 x 2 | 1.552(4) | P3-O28A | 1.555(8) |
|  |  | P2-O22 | 1.565(5) | P3-O31 | 1.515(6) |
|  |  | P2-O30 | 1.474(7) | **BVS P3** | **5.034** |
|  |  | **BVS P2** | **5.007** |  |  |

**Table S3:** Bond valence sums and bond distances for Cs_4.4_K_0.6_[(UO_2_)_6_O_4_F(PO_4_)_4_(UO_2_)] (**3**)._._

| Interaction | Distance | Interaction | Distance | Interaction | Distance |
| --- | --- | --- | --- | --- | --- |
| U1 – O1 | 1.795(5) | U2 – O3 | 2.298(5) | U3 – O3 | 2.353(5) |
| U1 – O2 | 1.801(5) | U2 – O4 | 2.404(5) | U3 – O6 | 2.280(5) |
| U1 – O3 | 2.231(5) | U2 – O5 | 2.390(5) | U3 – O7 | 2.351(5) |
| U1 – O4 | 2.611(6) | U2 – O6 | 2.333(4) | U3 – O8 | 2.295(6) |
| U1 – O5 | 2.602(6) | U2 – O9 | 1.785(5) | U3 – O12 | 1.794(6) |
| U1 – O6 | 2.245(4) | U2 – O10 | 1.780(5) | U3 – O13 | 1.783(5) |
| U1 – O7 | 2.534(5) | U2 – O11 | 2.351(5) | U3 – O14 | 2.355(5) |
| U1 – O8 | 2.728(7) | **U2 BVS** | **6.145** | **BVS U3** | **6.261** |
| **U1 BVS** | **6.003** | P1 – O7 | 1.540(5) | P2 – O4 | 1.541(5) |
| U4 – O15 | 1.774(9) | P1 – O8 | 1.531(6) | P2 – O5 | 1.537(6) |
| U4 – O16 | 1.755(10) | P1 – O11 | 1.520(5) | P2 – O14 | 1.514(5) |
| U4 – O17 x 2 | 2.278(7) | P1 – O17 | 1.517(6) | P2 – O18 | 1.512(6) |
| U4 – O18 x 2 | 2.293(6) | **BVS P1** | **5.103** | **BVS P2** | **5.119** |
| **BVS U4** | **6.020** |  |  |  |  |

**Table S4:** Bond valence sums and bond distances for Rb_4.4_K_0.6_[(UO_2_)_6_O_4_F(PO_4_)_4_(UO_2_)] (**4**).

| Interaction | Distance | Interaction | Distance | Interaction | Distance |
| --- | --- | --- | --- | --- | --- |
| U1 – O1 | 1.804(5) | U2 – O3 | 2.329(4) | U3 – O3 | 2.405(4) |
| U1 – O2 | 1.795(5) | U2 – O4 | 2.443(4) | U3 – O6 | 2.247(4) |
| U1 – O3 | 2.279(4) | U2 – O5 | 2.419(5) | U3 – O7 | 2.356(4) |
| U1 – O4 | 2.585(5) | U2 – O6 | 2.300(4) | U3 – O8 | 2.268(5) |
| U1 – O5 | 2.569(5) | U2 – O9 | 1.789(5) | U3 – O12 | 1.792(5) |
| U1 – O6 | 2.202(4) | U2 – O10 | 1.773(5) | U3 – O13 | 1.796(5) |
| U1 – O7 | 2.507(5) | U2 – O11 | 2.357(5) | U3 – O14 | 2.369(5) |
| U1 – O8 | 2.877(8) | **U2 BVS** | **6.086** | **BVS U3** | **6.228** |
| **U1 BVS** | **5.984** | P1 – O7 | 1.547(4) | P2 – O4 | 1.549(4) |
| U4 – O15 | 1.771(9) | P1 – O8 | 1.517(6) | P2 – O5 | 1.540(5) |
| U4 – O16 | 1.740(9) | P1 – O11 | 1.519(5) | P2 – O14 | 1.514(5) |
| U4 – O17 x 2 | 2.265(6) | P1 – O17 | 1.514(6) | P2 – O18 | 1.523(6) |
| U4 – O18 x 2 | 2.294(6) | **BVS P1** | **5.143** | **BVS P2** | **5.043** |
| **BVS U4** | **6.112** |  |  |  |  |


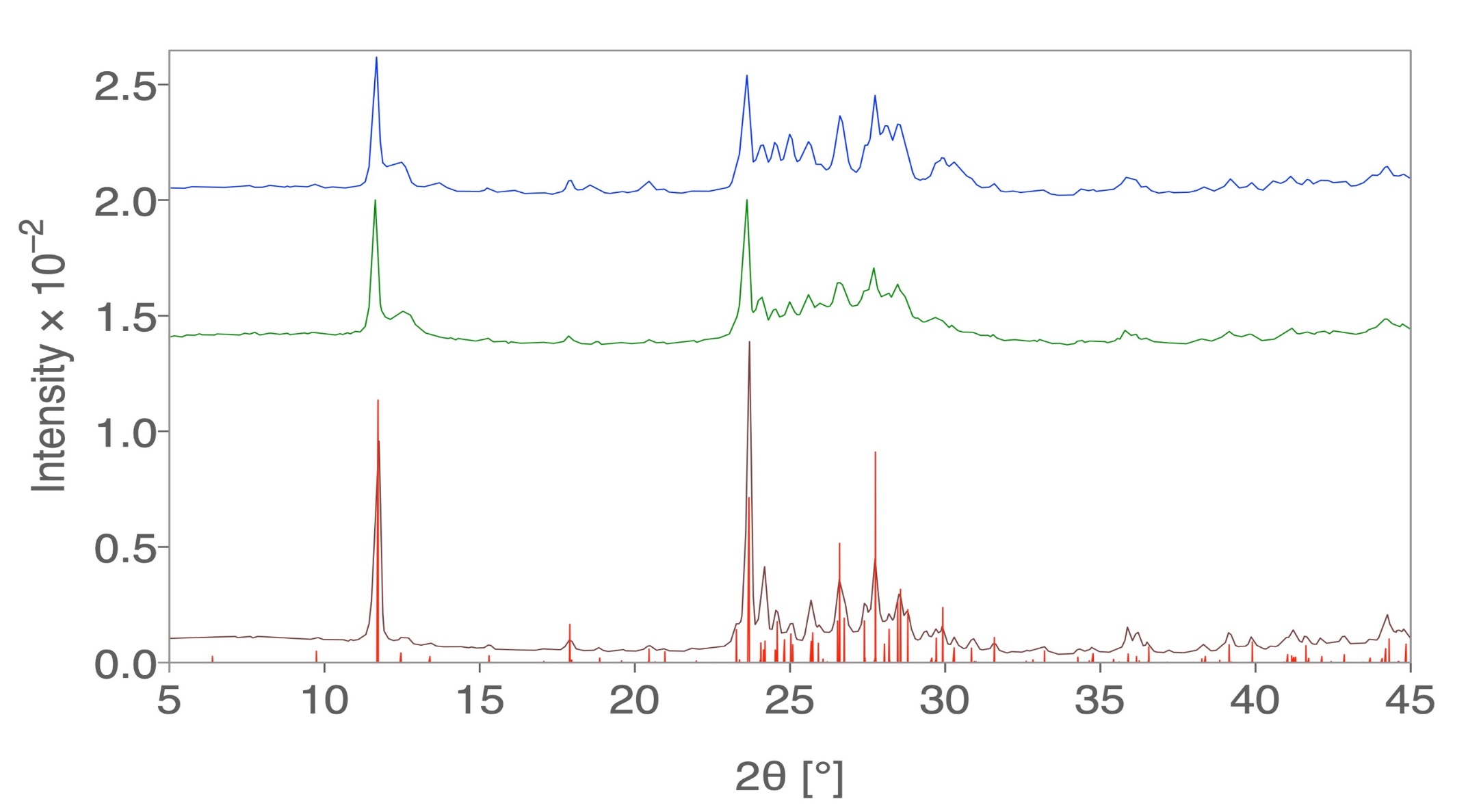


**Figure S1**. Powder X-ray diffraction patterns for Cs_11_[(UO_2_)_12_(PO_4_)_3_O_13_] (**1**) and the resulting powder ion exchange products. The pattern of phase pure Cs_11_[(UO_2_)_12_(PO_4_)_3_O_13_] is shown in black, the calculated pattern from the cif file is in red, the ion exchange product from soaking **1** in RbCl is shown in green, and the ion exchange product from soaking **1** in KCl is shown in blue.


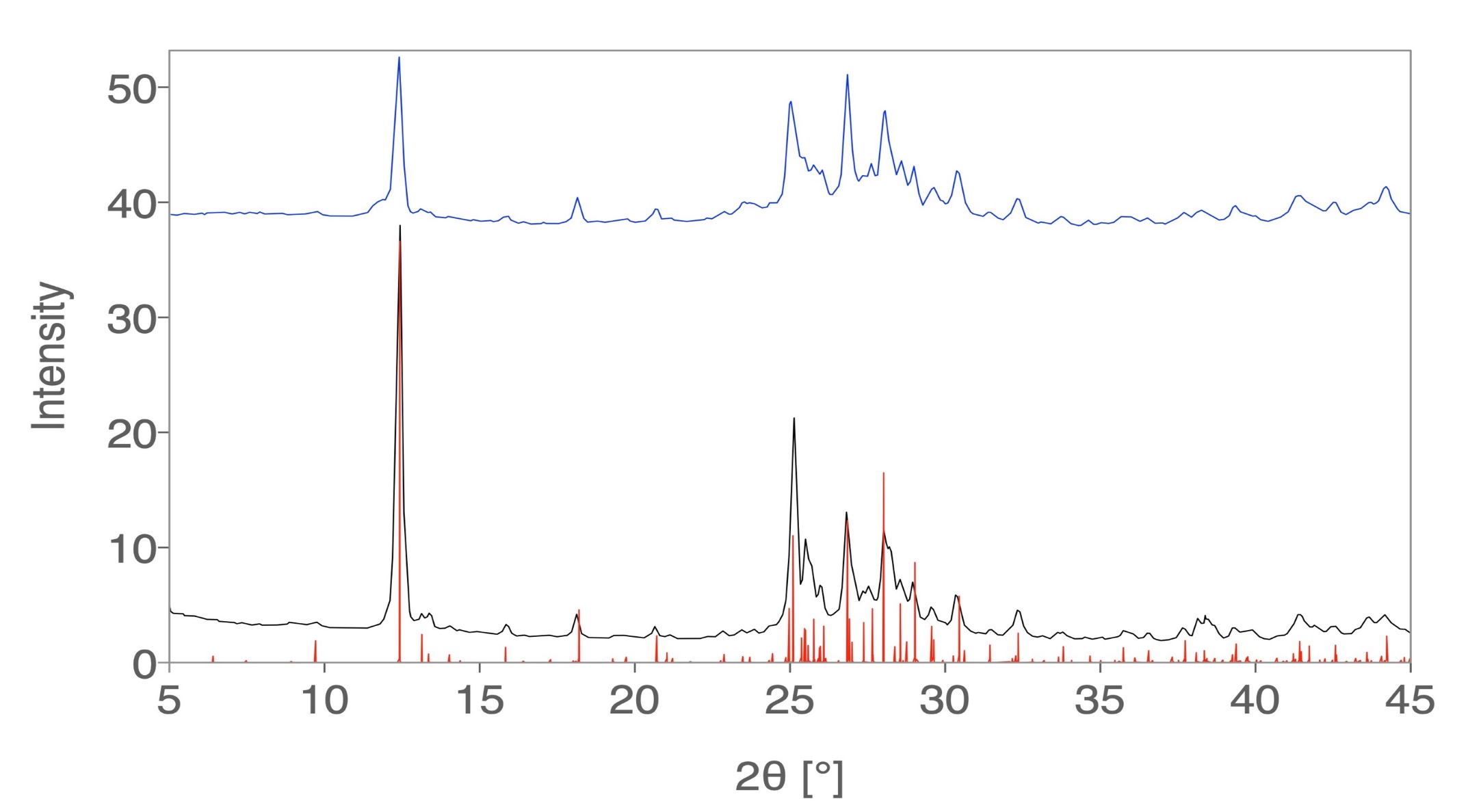


**Figure S2**. Powder X-ray diffraction patterns for Rb_11_[UO_2_)_12_(PO_4_)_3_O_12_F_2_] (**2**) and the resulting powder ion exchange product. The pattern of phase pure Rb_11_[UO_2_)_12_(PO_4_)_3_O_12_F_2_] is shown in black, the calculated pattern from the cif file is in red, and the ion exchange product from soaking **2** in CsCl is shown in blue.


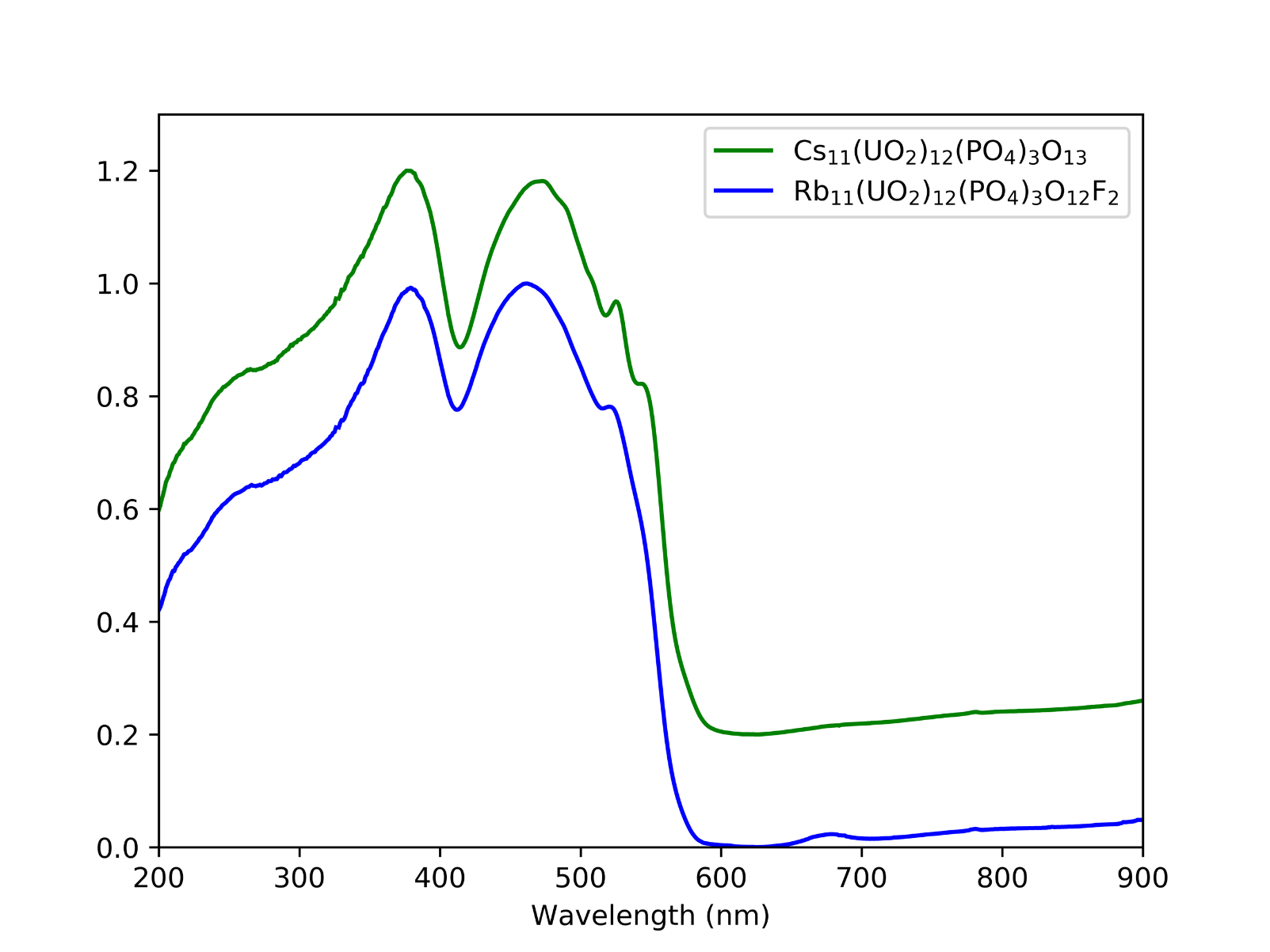


**Figure S3**. The UV-vis optical absorption spectra of Cs_11_[(UO_2_)_12_(PO_4_)_3_O_13_] (**1**) and

Rb_11_[UO_2_)_12_(PO_4_)_3_O_12_F_2_].


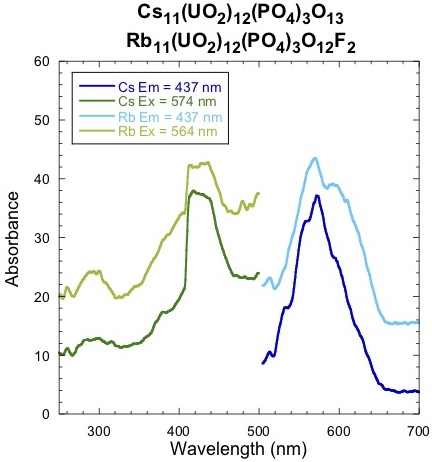


**Figure S4**. The fluorescence excitation and emission spectra of Cs_11_[(UO_2_)_12_(PO_4_)_3_O_13_] (**1**) and Rb_11_[UO_2_)_12_(PO_4_)_3_O_12_F_2_].


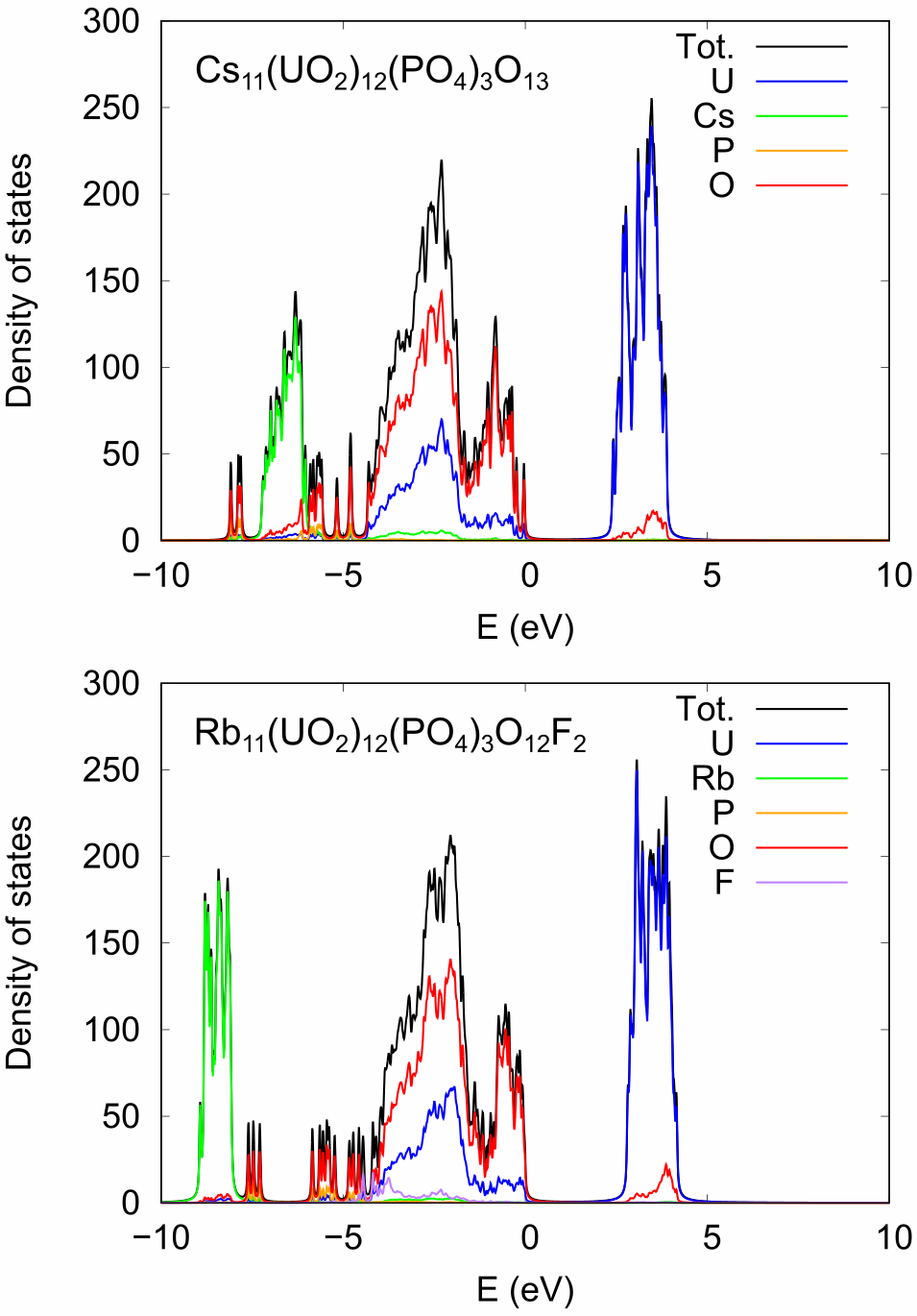


**Figure S5**. Density of states (DOS) and atom resolved projected DOS (PDOS) of the Cs_11_[(UO_2_)_12_(PO_4_)_3_O_13_] (up) and Rb_11_[UO_2_)_12_(PO_4_)_3_O_12_F_2_] (down) compounds. The PDOS of U, Cs/Rb, P, O, F and total DOS are shown in blue, green, orange, red, purple and black, respectively.
