# Supplementary material for: Observation of the Same New Sheet Topology in Both the Layered Uranyl Oxide-Phosphate Cs11[(UO2)12(PO4)3O13] and the Layered Uranyl Oxyfluoride-Phosphate Rb11[(UO2)12(PO4)3O12F2] Prepared by Flux Crystal Growth
Source: Front Chem. 2019 Aug 21;7:583. doi: 10.3389/fchem.2019.00583 (PMC6712084; doi:10.3389/fchem.2019.00583)

checkCIF/PLATON report

Structure factors have been supplied for datablock(s) Cs11P3U12O49

THIS REPORT IS FOR GUIDANCE ONLY. IF USED AS PART OF A REVIEW PROCEDURE FOR PUBLICATION, IT SHOULD NOT REPLACE THE EXPERTISE OF AN EXPERIENCED CRYSTALLOGRAPHIC REFEREE.

No syntax errors found. CIF dictionary Interpreting this report

Datablock: Cs11P3U12O49

Bond precision: P- O = 0.0060 A Wavelength=0.71073

Cell:	a=14.9561(3)	b=17.9663(4)	c=20.8520(4)
	alpha=90	beta=90	gamma=90
Temperature:	300 K		

	Calculated	Reported
Volume	5603.1(2)	5603.1(2)
Space group	P n m a	P n m a
Hall group	-P 2ac 2n	-P 2ac 2n
Moiety formula	Cs3.76 O196 P12 U48, 38.664(Cs), 0.772(Cs2)	0.25(Cs43.98 O196 P12 U48)
Sum formula	Cs43.97 O196 P12 U48	Cs11 O49 P3 U12
Mr	20776.87	5194.62
Dx,g cm-3	6.157	6.158
Z	1	4
Mu (mm-1)	41.772	41.774
F000	8582.2	8583.0
F000'	8122.04	
h,k,lmax	24,29,34	24,29,34
Nref	13952	13924
Tmin,Tmax	0.094,0.659	0.071,0.166
Tmin'	0.071	

Correction method= # Reported T Limits: Tmin=0.071 Tmax=0.166
AbsCorr = MULTI-SCAN

Data completeness= 0.998 Theta(max)= 36.355

R(reflections)= 0.0262(12324) wR2(reflections)= 0.0473(13924)

S = 1.111 Npar= 438

The following ALERTS were generated. Each ALERT has the format
test-name_ALERT_alert-type_alert-level.
Click on the hyperlinks for more details of the test.

Alert level A

PLAT972_ALERT_2_A Check Calcd Resid. Dens. 0.62A From U7 -5.15 eA-3

Author Response: All residual electron density peaks and holes are less than 10% of nearby heavy atom, U, and are acceptable for publication.

PLAT972_ALERT_2_A Check Calcd Resid. Dens. 0.69A From U3 -4.78 eA-3

Author Response: All residual electron density peaks and holes are less than 10% of nearby heavy atom, U, and are acceptable for publication.

Alert level B

PLAT971_ALERT_2_B Check Calcd Resid. Dens. 0.46A From U7 3.46 eA-3

Author Response: All residual electron density peaks and holes are less than 10% of nearby heavy atom, U, and are acceptable for publication.

PLAT971_ALERT_2_B Check Calcd Resid. Dens. 0.47A From U3 2.66 eA-3

Author Response: All residual electron density peaks and holes are less than 10% of nearby heavy atom, U, and are acceptable for publication.

PLAT971_ALERT_2_B Check Calcd Resid. Dens. 0.27A From U7 2.60 eA-3

Author Response: All residual electron density peaks and holes are less than 10% of nearby heavy atom, U, and are acceptable for publication.

PLAT971_ALERT_2_B Check Calcd Resid. Dens. 0.63A From U1 2.58 eA-3

Author Response: All residual electron density peaks and holes are less than 10% of nearby heavy atom, U, and are acceptable for publication.

PLAT972_ALERT_2_B Check Calcd Resid. Dens. 0.75A From U7 -3.26 eA-3

Author Response: All residual electron density peaks and holes are less than 10% of nearby heavy atom, U, and are acceptable for publication.

PLAT972_ALERT_2_B Check Calcd Resid. Dens. 0.66A From U6 -2.99 eA-3

Author Response: All residual electron density peaks and holes are less than 10% of nearby heavy atom, U, and are acceptable for publication.

PLAT974_ALERT_2_B Check Calcd Negative Resid. Density on U3 -1.82 eA-3

Author Response: All residual electron density peaks and holes are less than 10% of nearby heavy atom, U, and are acceptable for publication.

Alert level C

```
PLAT193_ALERT_1_C Cell and Diffraction Temperatures Differ by ....          1 Degree
PLAT220_ALERT_2_C Non-Solvent  Resd 1  O   Ueq(max)/Ueq(min) Range        3.8 Ratio
PLAT241_ALERT_2_C High   'MainMol' Ueq as Compared to Neighbors of         O8 Check
PLAT241_ALERT_2_C High   'MainMol' Ueq as Compared to Neighbors of         O9 Check
PLAT242_ALERT_2_C Low    'MainMol' Ueq as Compared to Neighbors of         U2 Check
PLAT242_ALERT_2_C Low    'MainMol' Ueq as Compared to Neighbors of         P2 Check
PLAT906_ALERT_3_C Large K Value in the Analysis of Variance ......      2.306 Check
PLAT910_ALERT_3_C Missing # of FCF Reflection(s) Below Theta(Min).          5 Note
PLAT911_ALERT_3_C Missing FCF Refl Between Thmin & STh/L=    0.600          2 Report
PLAT971_ALERT_2_C Check Calcd Resid. Dens.  0.71A   From U2              2.36 eA-3
```

Author Response: All residual electron density peaks and holes are less than 10% of nearby heavy atom, U, and are acceptable for publication.

PLAT971_ALERT_2_C Check Calcd Resid. Dens. 0.66A From Cs3 2.26 eA-3

Author Response: All residual electron density peaks and holes are less than 10% of nearby heavy atom, U, and are acceptable for publication.

PLAT971_ALERT_2_C Check Calcd Resid. Dens. 0.43A From U3 2.19 eA-3

Author Response: All residual electron density peaks and holes are less than 10% of nearby heavy atom, U, and are acceptable for publication.

PLAT971_ALERT_2_C Check Calcd Resid. Dens. 0.93A From Cs1B 1.89 eA-3

Author Response: All residual electron density peaks and holes are less than 10% of nearby heavy atom, U, and are acceptable for publication.

PLAT971_ALERT_2_C Check Calcd Resid. Dens. 0.89A From Cs1A 1.86 eA-3

Author Response: All residual electron density peaks and holes are less than 10% of nearby heavy atom, U, and are acceptable for publication.

PLAT971_ALERT_2_C Check Calcd Resid. Dens. 1.14A From U7 1.82 eA-3

Author Response: All residual electron density peaks and holes are less than 10% of nearby heavy atom, U, and are acceptable for publication.

PLAT971 ALERT 2 C Check Calcd Resid. Dens. 0.44A From Cs5 1.70 eA-3

Author Response: All residual electron density peaks and holes are less than 10% of nearby heavy atom, U, and are acceptable for publication.

PLAT971 ALERT 2 C Check Calcd Resid. Dens. 0.65A From U4 1.68 eA-3

Author Response: All residual electron density peaks and holes are less than 10% of nearby heavy atom, U, and are acceptable for publication.

PLAT971 ALERT 2 C Check Calcd Resid. Dens. 0.79A From U1 1.67 eA-3

Author Response: All residual electron density peaks and holes are less than 10% of nearby heavy atom, U, and are acceptable for publication.

PLAT971 ALERT 2 C Check Calcd Resid. Dens. 0.23A From U6 1.66 eA-3

Author Response: All residual electron density peaks and holes are less than 10% of nearby heavy atom, U, and are acceptable for publication.

PLAT971 ALERT 2 C Check Calcd Resid. Dens. 0.87A From Cs4B 1.66 eA-3

Author Response: All residual electron density peaks and holes are less than 10% of nearby heavy atom, U, and are acceptable for publication.

PLAT971 ALERT 2 C Check Calcd Resid. Dens. 0.90A From O6 1.64 eA-3

Author Response: All residual electron density peaks and holes are less than 10% of nearby heavy atom, U, and are acceptable for publication.

PLAT971 ALERT 2 C Check Calcd Resid. Dens. 0.63A From U7 1.62 eA-3

Author Response: All residual electron density peaks and holes are less than 10% of nearby heavy atom, U, and are acceptable for publication.

PLAT971 ALERT 2 C Check Calcd Resid. Dens. 0.63A From U7 1.62 eA-3

Author Response: All residual electron density peaks and holes are less than 10% of nearby heavy atom, U, and are acceptable for publication.

PLAT971 ALERT 2 C Check Calcd Resid. Dens. 0.66A From Cs4A 1.62 eA-3

Author Response: All residual electron density peaks and holes are less than 10% of nearby heavy atom, U, and are acceptable for publication.

PLAT971 ALERT 2 C Check Calcd Resid. Dens. 0.53A From Cs5 1.52 eA-3

Author Response: All residual electron density peaks and holes are less than 10% of nearby heavy atom, U, and are acceptable for publication.

PLAT972 ALERT 2 C Check Calcd Resid. Dens. 0.60A From Cs3 -2.23 eA-3

Author Response: All residual electron density peaks and holes are less than 10% of nearby heavy atom, U, and are acceptable for publication.

PLAT972 ALERT 2 C Check Calcd Resid. Dens. 0.35A From U7 -1.90 eA-3

Author Response: All residual electron density peaks and holes are less than 10% of nearby heavy atom, U, and are acceptable for publication.

PLAT972 ALERT 2 C Check Calcd Resid. Dens. 0.61A From U4 -1.86 eA-3

Author Response: All residual electron density peaks and holes are less than 10% of nearby heavy atom, U, and are acceptable for publication.

PLAT972 ALERT 2 C Check Calcd Resid. Dens. 0.76A From U6 -1.82 eA-3

Author Response: All residual electron density peaks and holes are less than 10% of nearby heavy atom, U, and are acceptable for publication.

PLAT972 ALERT 2 C Check Calcd Resid. Dens. 0.44A From P1 -1.78 eA-3

Author Response: All residual electron density peaks and holes are less than 10% of nearby heavy atom, U, and are acceptable for publication.

PLAT972 ALERT 2 C Check Calcd Resid. Dens. 0.23A From Cs3 -1.77 eA-3

Author Response: All residual electron density peaks and holes are less than 10% of nearby heavy atom, U, and are acceptable for publication.

PLAT972 ALERT 2 C Check Calcd Resid. Dens. 0.82A From Cs5 -1.69 eA-3

Author Response: All residual electron density peaks and holes are less than 10% of nearby heavy atom, U, and are acceptable for publication.

PLAT972 ALERT 2 C Check Calcd Resid. Dens. 0.51A From U1 -1.63 eA-3

Author Response: All residual electron density peaks and holes are less than 10% of nearby heavy atom, U, and are acceptable for publication.

```
PLAT972_ALERT_2_C Check Calcd Resid. Dens.  0.47A   From U2            -1.60 eA-3
```

Author Response: All residual electron density peaks and holes are less than 10% of nearby heavy atom, U, and are acceptable for publication.

```
PLAT972_ALERT_2_C Check Calcd Resid. Dens.  1.47A   From U4            -1.51 eA-3
```

Author Response: All residual electron density peaks and holes are less than 10% of nearby heavy atom, U, and are acceptable for publication.

```
PLAT975_ALERT_2_C Check Calcd Resid. Dens.  0.91A   From O26            1.40 eA-3
PLAT976_ALERT_2_C Check Calcd Resid. Dens.  1.07A   From O23           -1.37 eA-3
```

Alert level G

```
PLAT003_ALERT_2_G Number of Uiso or Uij Restrained non-H Atoms ...          2 Report
PLAT004_ALERT_5_G Polymeric Structure Found with Maximum Dimension          3 Info
PLAT012_ALERT_1_G No       _shelx_res_checksum Found in CIF ......     Please Check
PLAT042_ALERT_1_G Calc. and Reported MoietyFormula Strings  Differ     Please Check
PLAT045_ALERT_1_G Calculated and Reported Z Differ by a Factor ...       0.25 Check
PLAT068_ALERT_1_G Reported F000 Differs from Calcd (or Missing)...     Please Check
PLAT083_ALERT_2_G SHELXL Second Parameter in WGHT  Unusually Large      58.78 Why ?
PLAT186_ALERT_4_G The CIF-Embedded .res File Contains ISOR Records          1 Report
PLAT300_ALERT_4_G Atom Site Occupancy of P3         Constrained at        0.5 Check
PLAT300_ALERT_4_G Atom Site Occupancy of O23        Constrained at        0.5 Check
PLAT300_ALERT_4_G Atom Site Occupancy of O27        Constrained at        0.5 Check
PLAT300_ALERT_4_G Atom Site Occupancy of O28        Constrained at        0.5 Check
PLAT300_ALERT_4_G Atom Site Occupancy of O29        Constrained at        0.5 Check
PLAT301_ALERT_3_G Main Residue  Disorder .............(Resd  1  )          9% Note
PLAT302_ALERT_4_G Anion/Solvent/Minor-Residue Disorder (Resd  2  )        100% Note
PLAT302_ALERT_4_G Anion/Solvent/Minor-Residue Disorder (Resd  3  )        100% Note
PLAT302_ALERT_4_G Anion/Solvent/Minor-Residue Disorder (Resd  4  )        100% Note
PLAT302_ALERT_4_G Anion/Solvent/Minor-Residue Disorder (Resd  5  )        100% Note
PLAT302_ALERT_4_G Anion/Solvent/Minor-Residue Disorder (Resd  9  )        100% Note
PLAT302_ALERT_4_G Anion/Solvent/Minor-Residue Disorder (Resd 10  )        100% Note
PLAT794_ALERT_5_G Tentative Bond Valency for U1       (VI)      .        6.01 Info
PLAT794_ALERT_5_G Tentative Bond Valency for U2       (VI)      .        6.00 Info
PLAT794_ALERT_5_G Tentative Bond Valency for U3       (VI)      .        5.98 Info
PLAT794_ALERT_5_G Tentative Bond Valency for U4       (VI)      .        5.82 Info
PLAT794_ALERT_5_G Tentative Bond Valency for U5       (VI)      .        5.96 Info
PLAT860_ALERT_3_G Number of Least-Squares Restraints ............          14 Note
PLAT912_ALERT_4_G Missing # of FCF Reflections Above STh/L=  0.600         21 Note
PLAT913_ALERT_3_G Missing # of Very Strong Reflections in FCF ....          1 Note
PLAT933_ALERT_2_G Number of OMIT Records in Embedded .res File ...          2 Note
```

```
 2 ALERT level A = Most likely a serious problem - resolve or explain
 7 ALERT level B = A potentially serious problem, consider carefully
37 ALERT level C = Check. Ensure it is not caused by an omission or oversight
29 ALERT level G = General information/check it is not something unexpected

 5 ALERT type 1 CIF construction/syntax error, inconsistent or missing data
45 ALERT type 2 Indicator that the structure model may be wrong or deficient
 6 ALERT type 3 Indicator that the structure quality may be low
13 ALERT type 4 Improvement, methodology, query or suggestion
 6 ALERT type 5 Informative message, check
```

It is advisable to attempt to resolve as many as possible of the alerts in all categories. Often the minor alerts point to easily fixed oversights, errors and omissions in your CIF or refinement strategy, so attention to these fine details can be worthwhile. In order to resolve some of the more serious problems it may be necessary to carry out additional measurements or structure refinements. However, the purpose of your study may justify the reported deviations and the more serious of these should normally be commented upon in the discussion or experimental section of a paper or in the "special_details" fields of the CIF. checkCIF was carefully designed to identify outliers and unusual parameters, but every test has its limitations and alerts that are not important in a particular case may appear. Conversely, the absence of alerts does not guarantee there are no aspects of the results needing attention. It is up to the individual to critically assess their own results and, if necessary, seek expert advice.

Publication of your CIF in IUCr journals

A basic structural check has been run on your CIF. These basic checks will be run on all CIFs submitted for publication in IUCr journals (*Acta Crystallographica*, *Journal of Applied Crystallography*, *Journal of Synchrotron Radiation*); however, if you intend to submit to *Acta Crystallographica Section C* or *E* or *IUCrData*, you should make sure that full publication checks are run on the final version of your CIF prior to submission.

Publication of your CIF in other journals

Please refer to the *Notes for Authors* of the relevant journal for any special instructions relating to CIF submission.

PLATON version of 20/08/2018; check.def file version of 20/08/2018

Datablock Cs11P3U12O49 - ellipsoid plot

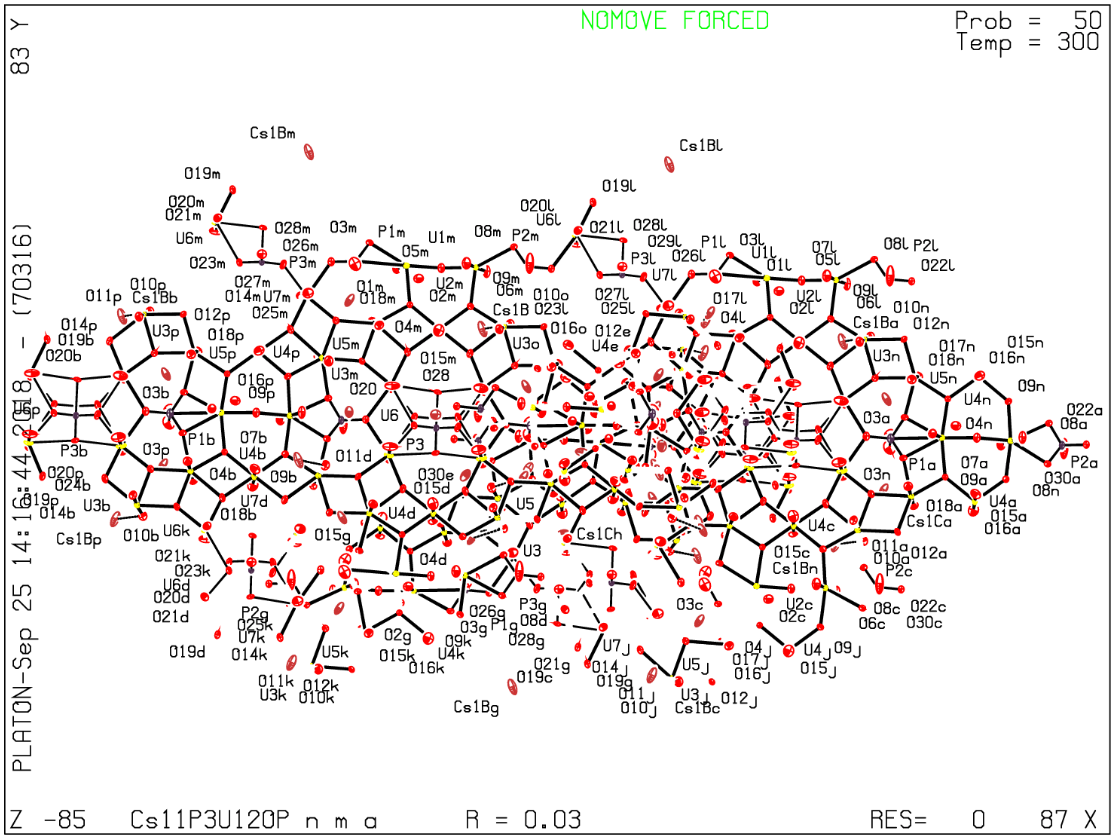

checkCIF/PLATON report

Structure factors have been supplied for datablock(s) Rb11U12P3O48F2

THIS REPORT IS FOR GUIDANCE ONLY. IF USED AS PART OF A REVIEW PROCEDURE FOR PUBLICATION, IT SHOULD NOT REPLACE THE EXPERTISE OF AN EXPERIENCED CRYSTALLOGRAPHIC REFEREE.

No syntax errors found. CIF dictionary Interpreting this report

Datablock: Rb11U12P3O48F2

```
Bond precision:  P- O = 0.0085 A          Wavelength=0.71073

Cell:            a=14.1258(3)     b=18.0121(4)     c=20.6241(4)
                 alpha=90         beta=90          gamma=90
Temperature:     301 K
```

	Calculated	Reported
Volume	5247.50(19)	5247.50(19)
Space group	P n m a	P n m a
Hall group	-P 2ac 2n	-P 2ac 2n
Moiety formula	F4 O96 P6 U24, 0.374(Rb2), 21.248(Rb)	0.25(F8 O192 P12 Rb44.01 U48)
Sum formula	F4 O96 P6 Rb22 U24	F2 O48 P3 Rb11 U12
Mr	9390.50	4695.65
Dx,g cm-3	5.943	5.944
Z	2	4
Mu (mm-1)	47.228	47.232
F000	7831.7	7832.0
F000'	7350.99	
h,k,lmax	23,30,34	23,30,34
Nref	13076	13063
Tmin,Tmax	0.073,0.624	0.073,0.166
Tmin'	0.047	

```
Correction method= # Reported T Limits: Tmin=0.073 Tmax=0.166
AbsCorr = MULTI-SCAN

Data completeness= 0.999          Theta(max)= 36.353

R(reflections)= 0.0278( 11301)    wR2(reflections)= 0.0551( 13063)

S = 1.128                   Npar= 418
```

The following ALERTS were generated. Each ALERT has the format
test-name_ALERT_alert-type_alert-level.
Click on the hyperlinks for more details of the test.

Alert level A

PLAT974_ALERT_2_A Check Calcd Negative Residual Density on Rb1B -2.17 eA-3

Author Response: The residual electron density is less than 10% of the heaviest atom in the structure, U, and splitting the site further did not result in a better solution.

Alert level B

PLAT971_ALERT_2_B Check Calcd Residual Density 0.66A From U7 3.27 eA-3

Author Response: The residual electron density is less than 10% of the heaviest atom in the structure, U, and splitting the site further did not result in a better solution.

PLAT971_ALERT_2_B Check Calcd Residual Density 0.65A From Rb4A 2.95 eA-3

Author Response: The residual electron density is less than 10% of the heaviest atom in the structure, U, and splitting the site further did not result in a better solution.

PLAT971_ALERT_2_B Check Calcd Residual Density 0.59A From Rb2 2.57 eA-3

Author Response: The residual electron density is less than 10% of the heaviest atom in the structure, U, and splitting the site further did not result in a better solution.

PLAT972_ALERT_2_B Check Calcd Residual Density 0.64A From Rb1A -2.72 eA-3

Author Response: The residual electron density is less than 10% of the heaviest atom in the structure, U, and splitting the site further did not result in a better solution.

PLAT972_ALERT_2_B Check Calcd Residual Density 0.48A From Rb4B -2.61 eA-3

Author Response: The residual electron density is less than 10% of the heaviest atom in the structure, U, and splitting the site further did not result in a better solution.

PLAT972_ALERT_2_B Check Calcd Residual Density 0.48A From Rb1A -2.52 eA-3

Author Response: The residual electron density is less than 10% of the heaviest atom in the structure, U, and splitting the site further did not result in a better solution.

Alert level C

```
PLAT220 ALERT 2 C Non-Solvent Resd 1   O   Ueq(max)/Ueq(min) Range        4.0 Ratio
PLAT241 ALERT 2 C High   'MainMol' Ueq as Compared to Neighbors of        F5 Check
PLAT242 ALERT 2 C Low    'MainMol' Ueq as Compared to Neighbors of        P2 Check
PLAT906 ALERT 3 C Large K value in the Analysis of Variance ......      2.616 Check
PLAT910 ALERT 3 C Missing # of FCF Reflection(s) Below Theta(Min).         5 Note
PLAT911 ALERT 3 C Missing # FCF Refl Between THmin & STh/L=  0.600         2 Report
PLAT971 ALERT 2 C Check Calcd Residual Density  0.63A From      U6       2.49 eA-3
```

Author Response: The residual electron density is less than 10% of the heaviest atom in the structure, U, and splitting the site further did not result in a better solution.

```
PLAT971 ALERT 2 C Check Calcd Residual Density  0.98A From    Rb1B       2.16 eA-3
```

Author Response: The residual electron density is less than 10% of the heaviest atom in the structure, U, and splitting the site further did not result in a better solution.

```
PLAT971 ALERT 2 C Check Calcd Residual Density  0.49A From    Rb4A       2.09 eA-3
```

Author Response: The residual electron density is less than 10% of the heaviest atom in the structure, U, and splitting the site further did not result in a better solution.

```
PLAT971 ALERT 2 C Check Calcd Residual Density  0.33A From    Rb4B       1.98 eA-3
```

Author Response: The residual electron density is less than 10% of the heaviest atom in the structure, U, and splitting the site further did not result in a better solution.

```
PLAT971 ALERT 2 C Check Calcd Residual Density  0.72A From      U5       1.91 eA-3
```

Author Response: The residual electron density is less than 10% of the heaviest atom in the structure, U, and splitting the site further did not result in a better solution.

```
PLAT971 ALERT 2 C Check Calcd Residual Density  0.73A From      U5       1.62 eA-3
```

Author Response: The residual electron density is less than 10% of the heaviest atom in the structure, U, and splitting the site further did not result in a better solution.

PLAT971 ALERT 2 C Check Calcd Residual Density 0.72A From U7 1.62 eA-3

Author Response: The residual electron density is less than 10% of the heaviest atom in the structure, U, and splitting the site further did not result in a better solution.

PLAT971 ALERT 2 C Check Calcd Residual Density 0.82A From U4 1.58 eA-3

Author Response: The residual electron density is less than 10% of the heaviest atom in the structure, U, and splitting the site further did not result in a better solution.

PLAT971 ALERT 2 C Check Calcd Residual Density 0.63A From U4 1.54 eA-3

Author Response: The residual electron density is less than 10% of the heaviest atom in the structure, U, and splitting the site further did not result in a better solution.

PLAT971 ALERT 2 C Check Calcd Residual Density 0.68A From U1 1.54 eA-3

Author Response: The residual electron density is less than 10% of the heaviest atom in the structure, U, and splitting the site further did not result in a better solution.

PLAT971 ALERT 2 C Check Calcd Residual Density 0.52A From U1 1.52 eA-3

Author Response: The residual electron density is less than 10% of the heaviest atom in the structure, U, and splitting the site further did not result in a better solution.

PLAT971 ALERT 2 C Check Calcd Residual Density 0.65A From Rb4B 1.52 eA-3

Author Response: The residual electron density is less than 10% of the heaviest atom in the structure, U, and splitting the site further did not result in a better solution.

PLAT972 ALERT 2 C Check Calcd Residual Density 0.47A From P1 -2.41 eA-3

Author Response: The residual electron density is less than 10% of the heaviest atom in the structure, U, and splitting the site further did not result in a better solution.

PLAT972 ALERT 2 C Check Calcd Residual Density 0.37A From P1 -2.29 eA-3

Author Response: The residual electron density is less than 10% of the heaviest atom in the structure, U, and splitting the site further did not result in a better solution.

PLAT972 ALERT 2 C Check Calcd Residual Density 0.39A From Rb1A -2.18 eA-3

Author Response: The residual electron density is less than 10% of the heaviest atom in the structure, U, and splitting the site further did not result in a better solution.

PLAT972 ALERT 2 C Check Calcd Residual Density 0.39A From Rb1A -2.18 eA-3

Author Response: The residual electron density is less than 10% of the heaviest atom in the structure, U, and splitting the site further did not result in a better solution.

PLAT972 ALERT 2 C Check Calcd Residual Density 0.49A From P1 -2.18 eA-3

Author Response: The residual electron density is less than 10% of the heaviest atom in the structure, U, and splitting the site further did not result in a better solution.

PLAT972 ALERT 2 C Check Calcd Residual Density 1.16A From O27 -1.98 eA-3

Author Response: The residual electron density is less than 10% of the heaviest atom in the structure, U, and splitting the site further did not result in a better solution.

PLAT972 ALERT 2 C Check Calcd Residual Density 1.19A From O3 -1.98 eA-3

Author Response: The residual electron density is less than 10% of the heaviest atom in the structure, U, and splitting the site further did not result in a better solution.

PLAT972 ALERT 2 C Check Calcd Residual Density 1.18A From U6 -1.78 eA-3

Author Response: The residual electron density is less than 10% of the heaviest atom in the structure, U, and splitting the site further did not result in a better solution.

PLAT972 ALERT 2 C Check Calcd Residual Density 1.28A From O4 -1.78 eA-3

Author Response: The residual electron density is less than 10% of the heaviest atom in the structure, U, and splitting the site further did not result in a better solution.

PLAT972 ALERT 2 C Check Calcd Residual Density 0.52A From Rb4A -1.71 eA-3

Author Response: The residual electron density is less than 10% of the heaviest atom in the structure, U, and splitting the site further did not result in a better solution.

PLAT972 ALERT 2 C Check Calcd Residual Density 0.97A From O6 -1.68 eA-3

Author Response: The residual electron density is less than 10% of the heaviest atom in the structure, U, and splitting the site further did not result in a better solution.

PLAT972 ALERT 2 C Check Calcd Residual Density 0.61A From Rb2 -1.64 eA-3

Author Response: The residual electron density is less than 10% of the heaviest atom in the structure, U, and splitting the site further did not result in a better solution.

PLAT972 ALERT 2 C Check Calcd Residual Density 0.63A From U5 -1.64 eA-3

Author Response: The residual electron density is less than 10% of the heaviest atom in the structure, U, and splitting the site further did not result in a better solution.

PLAT972 ALERT 2 C Check Calcd Residual Density 0.60A From U3 -1.64 eA-3

Author Response: The residual electron density is less than 10% of the heaviest atom in the structure, U, and splitting the site further did not result in a better solution.

PLAT972 ALERT 2 C Check Calcd Residual Density 0.49A From U1 -1.62 eA-3

Author Response: The residual electron density is less than 10% of the heaviest atom in the structure, U, and splitting the site further did not result in a better solution.

Alert level G

```
PLAT004 ALERT 5 G Polymeric Structure Found with Maximum Dimension          3 Info
PLAT012 ALERT 1 G No     _shelx_res_checksum found in CIF ........     Please Check
PLAT042 ALERT 1 G Calc. and Reported MoietyFormula Strings  Differ     Please Check
PLAT045 ALERT 1 G Calculated and Reported Z Differ by a Factor ...       0.50 Check
PLAT068 ALERT 1 G Reported F000 Differs from Calcd (or Missing)...     Please Check
PLAT083 ALERT 2 G SHELXL Second Parameter in WGHT  Unusually Large      51.51 Why ?
PLAT171 ALERT 4 G The CIF-Embedded .res File Contains EADP Records          3 Report
PLAT300 ALERT 4 G Atom Site Occupancy of P3      is Constrained at        0.5 Check
PLAT300 ALERT 4 G Atom Site Occupancy of F28B    is Constrained at        0.5 Check
PLAT300 ALERT 4 G Atom Site Occupancy of O28A    is Constrained at        0.5 Check
PLAT301 ALERT 3 G Main Residue  Disorder .............(Resd  1)..         4% Note
PLAT302 ALERT 4 G Anion/Solvent/Minor-Residue Disorder (Resd  2)..       100% Note
PLAT302 ALERT 4 G Anion/Solvent/Minor-Residue Disorder (Resd  3)..       100% Note
PLAT302 ALERT 4 G Anion/Solvent/Minor-Residue Disorder (Resd  5)..       100% Note
PLAT302 ALERT 4 G Anion/Solvent/Minor-Residue Disorder (Resd  6)..       100% Note
PLAT302 ALERT 4 G Anion/Solvent/Minor-Residue Disorder (Resd  9)..       100% Note
PLAT302 ALERT 4 G Anion/Solvent/Minor-Residue Disorder (Resd 10)..       100% Note
PLAT302 ALERT 4 G Anion/Solvent/Minor-Residue Disorder (Resd 11)..       100% Note
PLAT302 ALERT 4 G Anion/Solvent/Minor-Residue Disorder (Resd 12)..       100% Note
```

```
PLAT302 ALERT 4 G Anion/Solvent/Minor-Residue Disorder (Resd 13)..       100% Note
PLAT794 ALERT 5 G Tentative Bond Valency for U3      (VI)    .....       6.01 Info
PLAT794 ALERT 5 G Tentative Bond Valency for U5      (VI)    .....       6.07 Info
PLAT860 ALERT 3 G Number of Least-Squares Restraints ............           1 Note
PLAT912 ALERT 4 G Missing # of FCF Reflections Above STh/L=  0.600          7 Note
PLAT913 ALERT 3 G Missing # of Very Strong Reflections in FCF ....          2 Note
PLAT933 ALERT 2 G Number of OMIT Records in Embedded .res File ...          2 Note
```

```
 1 ALERT level A = Most likely a serious problem - resolve or explain
 6 ALERT level B = A potentially serious problem, consider carefully
33 ALERT level C = Check. Ensure it is not caused by an omission or oversight
26 ALERT level G = General information/check it is not something unexpected

 4 ALERT type 1 CIF construction/syntax error, inconsistent or missing data
39 ALERT type 2 Indicator that the structure model may be wrong or deficient
 6 ALERT type 3 Indicator that the structure quality may be low
14 ALERT type 4 Improvement, methodology, query or suggestion
 3 ALERT type 5 Informative message, check
```

It is advisable to attempt to resolve as many as possible of the alerts in all categories. Often the minor alerts point to easily fixed oversights, errors and omissions in your CIF or refinement strategy, so attention to these fine details can be worthwhile. In order to resolve some of the more serious problems it may be necessary to carry out additional measurements or structure refinements. However, the purpose of your study may justify the reported deviations and the more serious of these should normally be commented upon in the discussion or experimental section of a paper or in the "special_details" fields of the CIF. checkCIF was carefully designed to identify outliers and unusual parameters, but every test has its limitations and alerts that are not important in a particular case may appear. Conversely, the absence of alerts does not guarantee there are no aspects of the results needing attention. It is up to the individual to critically assess their own results and, if necessary, seek expert advice.

Publication of your CIF in IUCr journals

A basic structural check has been run on your CIF. These basic checks will be run on all CIFs submitted for publication in IUCr journals (*Acta Crystallographica*, *Journal of Applied Crystallography*, *Journal of Synchrotron Radiation*); however, if you intend to submit to *Acta Crystallographica Section C* or *E* or *IUCrData*, you should make sure that full publication checks are run on the final version of your CIF prior to submission.

Publication of your CIF in other journals

Please refer to the *Notes for Authors* of the relevant journal for any special instructions relating to CIF submission.

PLATON version of 13/08/2017; check.def file version of 27/07/2017

Datablock Rb11U12P3O48F2 - ellipsoid plot

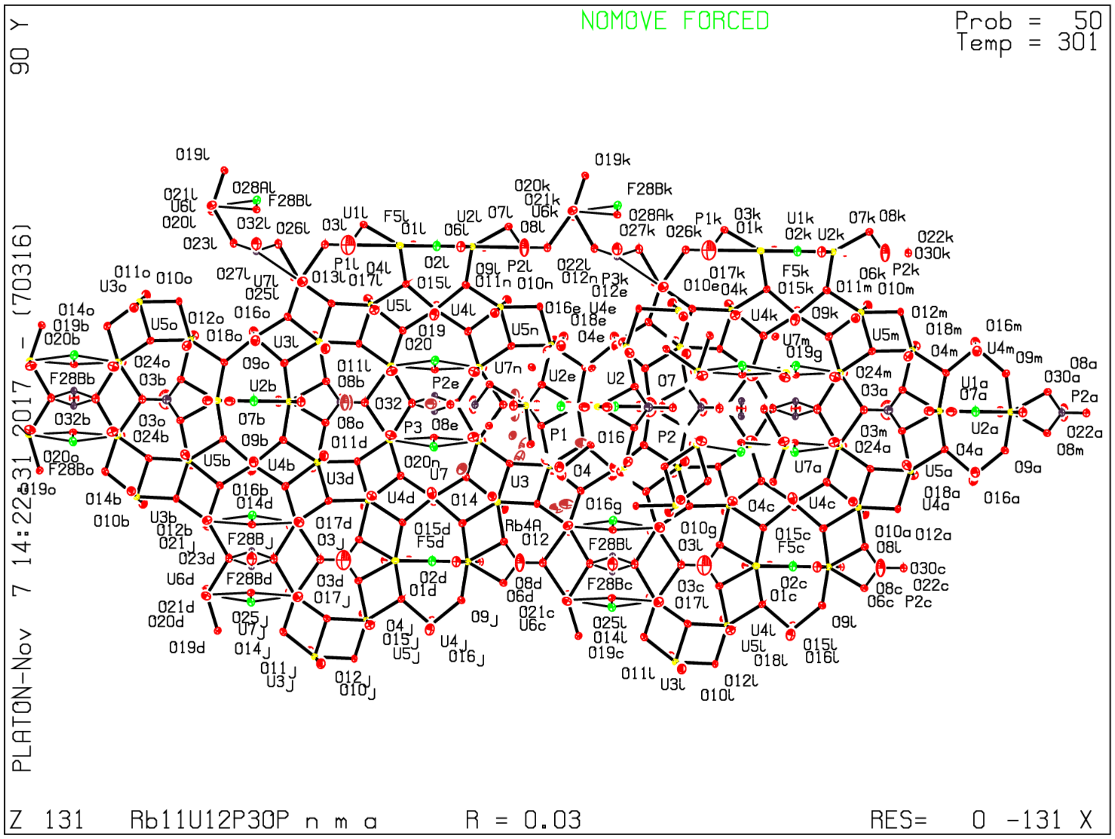

checkCIF/PLATON report

Structure factors have been supplied for datablock(s) Cs4KU7P4O34F

THIS REPORT IS FOR GUIDANCE ONLY. IF USED AS PART OF A REVIEW PROCEDURE FOR PUBLICATION, IT SHOULD NOT REPLACE THE EXPERTISE OF AN EXPERIENCED CRYSTALLOGRAPHIC REFEREE.

No syntax errors found. CIF dictionary Interpreting this report

Datablock: Cs4KU7P4O34F

Bond precision: P- O = 0.0060 A Wavelength=0.71073

Cell:	a=25.8529(6)	b=28.9285(6)	c=9.2321(2)
	alpha=90	beta=90	gamma=90
Temperature:	300 K		

	Calculated	Reported
Volume	6904.6(3)	6904.6(3)
Space group	F d d 2	F d d 2
Hall group	F 2 -2d	F 2 -2d
Moiety formula	F4 O132 P16 U28, 17.556(Cs), 2.444(K)	Cs4.39 F2 K0.61 O32 P4 U7
Sum formula	Cs17.56 F4 K2.44 O132 P16 U28	Cs4.39 F2 K0.61 O32 P4 U7
Mr	11777.29	2947.30
Dx,g cm-3	5.665	5.671
Z	2	8
Mu (mm-1)	37.647	37.649
F000	9840.0	9848.0
F000'	9310.85	
h,k,lmax	43,48,15	43,48,15
Nref	8400[4405]	8388
Tmin,Tmax	0.179,0.686	0.088,0.166
Tmin'	0.096	

Correction method= # Reported T Limits: Tmin=0.088 Tmax=0.166
AbsCorr = MULTI-SCAN

Data completeness= 1.90/1.00 Theta(max)= 36.355

R(reflections)= 0.0191(8243) wR2(reflections)= 0.0379(8388)

S = 1.141 Npar= 234

The following ALERTS were generated. Each ALERT has the format
test-name_ALERT_alert-type_alert-level.
Click on the hyperlinks for more details of the test.

Alert level C

```
STRVA01_ALERT_4_C           Flack test results are ambiguous.
           From the CIF: _refine_ls_abs_structure_Flack    0.414
           From the CIF: _refine_ls_abs_structure_Flack_su    0.006
PLAT041_ALERT_1_C Calc. and Reported SumFormula    Strings  Differ     Please Check
PLAT068_ALERT_1_C Reported F000 Differs from Calcd (or Missing)...     Please Check
PLAT077_ALERT_4_C Unitcell Contains Non-integer Number of Atoms ..     Please Check
PLAT241_ALERT_2_C High   'MainMol' Ueq as Compared to Neighbors of        O8 Check
PLAT241_ALERT_2_C High   'MainMol' Ueq as Compared to Neighbors of       O17 Check
PLAT241_ALERT_2_C High   'MainMol' Ueq as Compared to Neighbors of       O18 Check
PLAT242_ALERT_2_C Low    'MainMol' Ueq as Compared to Neighbors of        U3 Check
PLAT242_ALERT_2_C Low    'MainMol' Ueq as Compared to Neighbors of        U4 Check
PLAT242_ALERT_2_C Low    'MainMol' Ueq as Compared to Neighbors of        P1 Check
PLAT242_ALERT_2_C Low    'MainMol' Ueq as Compared to Neighbors of        P2 Check
PLAT911_ALERT_3_C Missing FCF Refl Between Thmin & STh/L=    0.600         3 Report
PLAT971_ALERT_2_C Check Calcd Resid. Dens.  0.76A   From Cs2          1.91 eA-3
PLAT971_ALERT_2_C Check Calcd Resid. Dens.  0.40A   From Cs2          1.65 eA-3
PLAT972_ALERT_2_C Check Calcd Resid. Dens.  0.86A   From Cs2         -2.43 eA-3
PLAT972_ALERT_2_C Check Calcd Resid. Dens.  0.74A   From U4          -2.11 eA-3
PLAT974_ALERT_2_C Check Calcd Negative Resid. Density on      Cs3A      -1.31 eA-3
PLAT975_ALERT_2_C Check Calcd Resid. Dens.  0.68A   From O4           0.86 eA-3
PLAT975_ALERT_2_C Check Calcd Resid. Dens.  1.04A   From O7           0.85 eA-3
```

Alert level G

```
FORMU01_ALERT_2_G  There is a discrepancy between the atom counts in the
            _chemical_formula_sum and the formula from the _atom_site* data.
            Atom count from _chemical_formula_sum:Cs4.39 F2 K0.61 O32 P4 U7
            Atom count from the _atom_site data:  Cs4.389 F1 K0.611 O33 P4 U7
CELLZ01_ALERT_1_G Difference between formula and atom_site contents detected.
CELLZ01_ALERT_1_G ALERT: Large difference may be due to a
            symmetry error - see SYMMG tests
           From the CIF: _cell_formula_units_Z    8
           From the CIF: _chemical_formula_sum  Cs4.39 F2 K0.61 O32 P4 U7
           TEST: Compare cell contents of formula and atom_site data

           atom    Z*formula  cif sites diff
           Cs        35.12     35.11    0.01
           F         16.00      8.00    8.00
           K          4.88      4.89   -0.01
           O        256.00    264.00   -8.00
           P         32.00     32.00    0.00
           U         56.00     56.00    0.00
PLAT004_ALERT_5_G Polymeric Structure Found with Maximum Dimension         1 Info
PLAT012_ALERT_1_G No       _shelx_res_checksum Found in CIF ......     Please Check
PLAT042_ALERT_1_G Calc. and Reported MoietyFormula Strings  Differ     Please Check
PLAT045_ALERT_1_G Calculated and Reported Z Differ by a Factor ...      0.25 Check
PLAT083_ALERT_2_G SHELXL Second Parameter in WGHT  Unusually Large     174.92 Why ?
PLAT171_ALERT_4_G The CIF-Embedded .res File Contains EADP Records         3 Report
PLAT300_ALERT_4_G Atom Site Occupancy of F3         Constrained at       0.5 Check
PLAT300_ALERT_4_G Atom Site Occupancy of O3         Constrained at       0.5 Check
PLAT301_ALERT_3_G Main Residue  Disorder .............(Resd  1  )        2% Note
PLAT302_ALERT_4_G Anion/Solvent/Minor-Residue Disorder (Resd  3  )      100% Note
PLAT302_ALERT_4_G Anion/Solvent/Minor-Residue Disorder (Resd  4  )      100% Note
PLAT302_ALERT_4_G Anion/Solvent/Minor-Residue Disorder (Resd  5  )      100% Note
PLAT302_ALERT_4_G Anion/Solvent/Minor-Residue Disorder (Resd  6  )      100% Note
```

```
PLAT794 ALERT 5 G Tentative Bond Valency for U4        (VI)      .       6.03 Info
PLAT910 ALERT 3 G Missing # of FCF Reflection(s) Below Theta(Min).          1 Note
PLAT912 ALERT 4 G Missing # of FCF Reflections Above STh/L=  0.600          4 Note
PLAT913 ALERT 3 G Missing # of Very Strong Reflections in FCF ....          1 Note
PLAT933 ALERT 2 G Number of OMIT Records in Embedded .res File ...          2 Note
```

```
  0 ALERT level A = Most likely a serious problem - resolve or explain
  0 ALERT level B = A potentially serious problem, consider carefully
 19 ALERT level C = Check. Ensure it is not caused by an omission or oversight
 21 ALERT level G = General information/check it is not something unexpected

  7 ALERT type 1 CIF construction/syntax error, inconsistent or missing data
 17 ALERT type 2 Indicator that the structure model may be wrong or deficient
  4 ALERT type 3 Indicator that the structure quality may be low
 10 ALERT type 4 Improvement, methodology, query or suggestion
  2 ALERT type 5 Informative message, check
```

It is advisable to attempt to resolve as many as possible of the alerts in all categories. Often the minor alerts point to easily fixed oversights, errors and omissions in your CIF or refinement strategy, so attention to these fine details can be worthwhile. In order to resolve some of the more serious problems it may be necessary to carry out additional measurements or structure refinements. However, the purpose of your study may justify the reported deviations and the more serious of these should normally be commented upon in the discussion or experimental section of a paper or in the "special_details" fields of the CIF. checkCIF was carefully designed to identify outliers and unusual parameters, but every test has its limitations and alerts that are not important in a particular case may appear. Conversely, the absence of alerts does not guarantee there are no aspects of the results needing attention. It is up to the individual to critically assess their own results and, if necessary, seek expert advice.

Publication of your CIF in IUCr journals

A basic structural check has been run on your CIF. These basic checks will be run on all CIFs submitted for publication in IUCr journals (*Acta Crystallographica*, *Journal of Applied Crystallography*, *Journal of Synchrotron Radiation*); however, if you intend to submit to *Acta Crystallographica Section C* or *E* or *IUCrData*, you should make sure that full publication checks are run on the final version of your CIF prior to submission.

Publication of your CIF in other journals

Please refer to the *Notes for Authors* of the relevant journal for any special instructions relating to CIF submission.

PLATON version of 19/10/2018; check.def file version of 15/10/2018

Datablock Cs4KU7P4O34F - ellipsoid plot

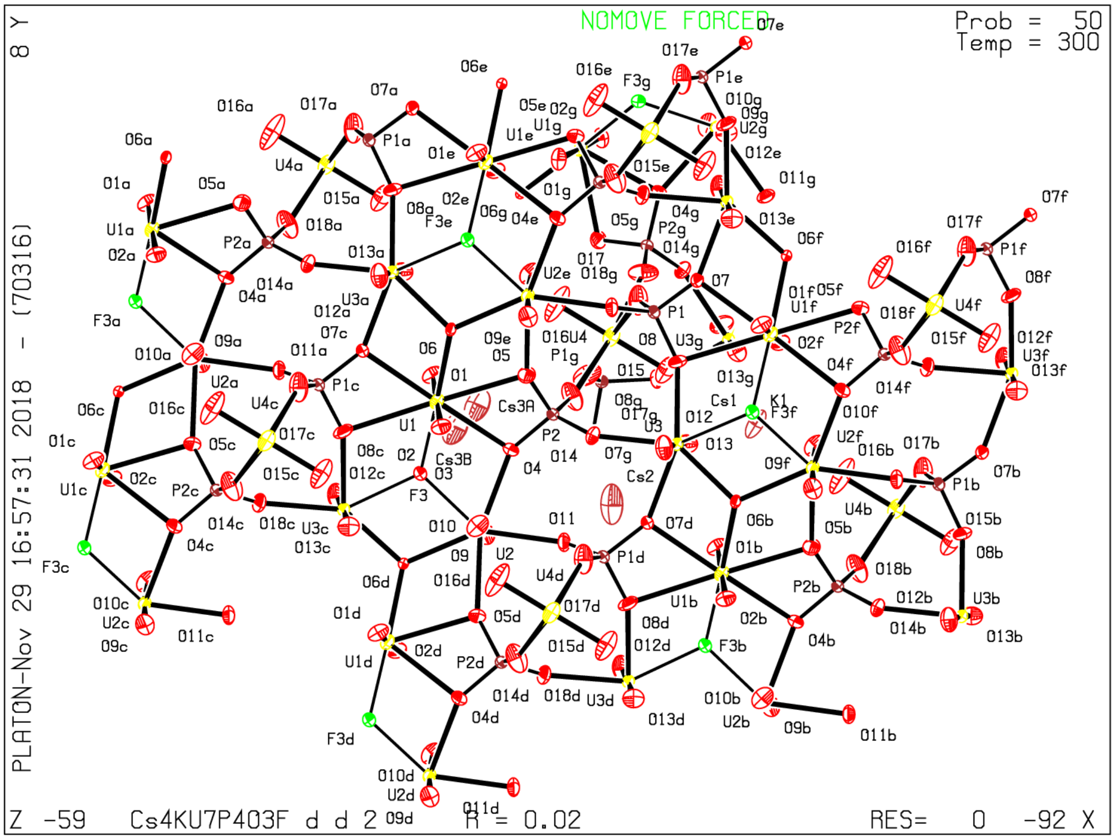

checkCIF/PLATON report

Structure factors have been supplied for datablock(s) Rb4KU7O34F

THIS REPORT IS FOR GUIDANCE ONLY. IF USED AS PART OF A REVIEW PROCEDURE FOR PUBLICATION, IT SHOULD NOT REPLACE THE EXPERTISE OF AN EXPERIENCED CRYSTALLOGRAPHIC REFEREE.

No syntax errors found. CIF dictionary Interpreting this report

Datablock: Rb4KU7O34F

```
Bond precision:  P- O = 0.0056 A         Wavelength=0.71073

Cell:             a=25.6593(5)      b=27.5792(5)      c=9.2591(2)
                  alpha=90          beta=90           gamma=90
Temperature:      300 K
```

	Calculated	Reported
Volume	6552.3(2)	6552.3(2)
Space group	F d d 2	F d d 2
Hall group	F 2 -2d	F 2 -2d
Moiety formula	F4 O132 P16 U28, 17.652(Rb), 2.348(K)	0.13(F8 K4.7 O264 P32 Rb35.3 U56)
Sum formula	F4 K2.35 O132 P16 Rb17.65 U28	F K0.59 O33 P4 Rb4.41 U7
Mr	10948.88	2737.20
Dx,g cm-3	5.550	5.549
Z	2	8
Mu (mm-1)	41.375	41.374
F000	9211.5	9211.0
F000'	8665.45	
h,k,lmax	42,45,15	42,45,15
Nref	7950[4168]	7945
Tmin,Tmax	0.385,0.661	0.073,0.166
Tmin'	0.121	

```
Correction method= # Reported T Limits: Tmin=0.073 Tmax=0.166
AbsCorr = MULTI-SCAN

Data completeness= 1.91/1.00       Theta(max)= 36.343

R(reflections)= 0.0179( 7715)      wR2(reflections)= 0.0402( 7945)

S = 1.081                    Npar= 234
```

The following ALERTS were generated. Each ALERT has the format
test-name_ALERT_alert-type_alert-level.
Click on the hyperlinks for more details of the test.

Alert level C

```
PLAT077_ALERT_4_C Unitcell Contains Non-integer Number of Atoms ..     Please Check
PLAT213_ALERT_2_C Atom O8              has ADP max/min Ratio .....      3.3 prolat
PLAT241_ALERT_2_C High   'MainMol' Ueq as Compared to Neighbors of       O8 Check
PLAT241_ALERT_2_C High   'MainMol' Ueq as Compared to Neighbors of      O17 Check
PLAT241_ALERT_2_C High   'MainMol' Ueq as Compared to Neighbors of      O18 Check
PLAT242_ALERT_2_C Low    'MainMol' Ueq as Compared to Neighbors of       U3 Check
PLAT242_ALERT_2_C Low    'MainMol' Ueq as Compared to Neighbors of       U4 Check
PLAT242_ALERT_2_C Low    'MainMol' Ueq as Compared to Neighbors of       P1 Check
PLAT971_ALERT_2_C Check Calcd Resid. Dens.  0.71A   From U2            1.76 eA-3
PLAT971_ALERT_2_C Check Calcd Resid. Dens.  0.69A   From Rb3B          1.52 eA-3
PLAT972_ALERT_2_C Check Calcd Resid. Dens.  0.67A   From Rb3A         -2.13 eA-3
PLAT972_ALERT_2_C Check Calcd Resid. Dens.  0.32A   From Rb3A         -1.97 eA-3
PLAT972_ALERT_2_C Check Calcd Resid. Dens.  0.33A   From Rb3B         -1.96 eA-3
PLAT972_ALERT_2_C Check Calcd Resid. Dens.  0.49A   From Rb3B         -1.82 eA-3
PLAT972_ALERT_2_C Check Calcd Resid. Dens.  0.46A   From U4           -1.64 eA-3
PLAT972_ALERT_2_C Check Calcd Resid. Dens.  0.46A   From U4           -1.57 eA-3
PLAT975_ALERT_2_C Check Calcd Resid. Dens.  0.90A   From O1            0.59 eA-3
PLAT975_ALERT_2_C Check Calcd Resid. Dens.  0.75A   From O8            0.58 eA-3
PLAT975_ALERT_2_C Check Calcd Resid. Dens.  0.95A   From O10           0.56 eA-3
```

Alert level G

```
FORMU01_ALERT_1_G  There is a discrepancy between the atom counts in the
            _chemical_formula_sum and _chemical_formula_moiety. This is
            usually due to the moiety formula being in the wrong format.
            Atom count from _chemical_formula_sum:   F1 K0.59 O33 P4 Rb4.41 U7
            Atom count from _chemical_formula_moiety:F1.04 K0.611 O34.32 P4.16 R
PLAT004_ALERT_5_G Polymeric Structure Found with Maximum Dimension        1 Info
PLAT012_ALERT_1_G No       _shelx_res_checksum Found in CIF ......     Please Check
PLAT042_ALERT_1_G Calc. and Reported MoietyFormula Strings  Differ     Please Check
PLAT045_ALERT_1_G Calculated and Reported Z Differ by a Factor ...       0.25 Check
PLAT068_ALERT_1_G Reported F000 Differs from Calcd (or Missing)...     Please Check
PLAT083_ALERT_2_G SHELXL Second Parameter in WGHT  Unusually Large      96.78 Why ?
PLAT171_ALERT_4_G The CIF-Embedded .res File Contains EADP Records        3 Report
PLAT300_ALERT_4_G Atom Site Occupancy of F3         Constrained at      0.5 Check
PLAT300_ALERT_4_G Atom Site Occupancy of O3         Constrained at      0.5 Check
PLAT301_ALERT_3_G Main Residue  Disorder .............(Resd  1  )        2% Note
PLAT302_ALERT_4_G Anion/Solvent/Minor-Residue Disorder (Resd  3  )      100% Note
PLAT302_ALERT_4_G Anion/Solvent/Minor-Residue Disorder (Resd  4  )      100% Note
PLAT302_ALERT_4_G Anion/Solvent/Minor-Residue Disorder (Resd  5  )      100% Note
PLAT302_ALERT_4_G Anion/Solvent/Minor-Residue Disorder (Resd  6  )      100% Note
PLAT794_ALERT_5_G Tentative Bond Valency for U4        (VI)      .      6.13 Info
PLAT910_ALERT_3_G Missing # of FCF Reflection(s) Below Theta(Min).        1 Note
PLAT933_ALERT_2_G Number of OMIT Records in Embedded .res File ...        2 Note
```

---

0 **ALERT level A** = Most likely a serious problem - resolve or explain
0 **ALERT level B** = A potentially serious problem, consider carefully
19 **ALERT level C** = Check. Ensure it is not caused by an omission or oversight
18 **ALERT level G** = General information/check it is not something unexpected

5 ALERT type 1 CIF construction/syntax error, inconsistent or missing data

```
20 ALERT type 2 Indicator that the structure model may be wrong or deficient
 2 ALERT type 3 Indicator that the structure quality may be low
 8 ALERT type 4 Improvement, methodology, query or suggestion
 2 ALERT type 5 Informative message, check
```

It is advisable to attempt to resolve as many as possible of the alerts in all categories. Often the minor alerts point to easily fixed oversights, errors and omissions in your CIF or refinement strategy, so attention to these fine details can be worthwhile. In order to resolve some of the more serious problems it may be necessary to carry out additional measurements or structure refinements. However, the purpose of your study may justify the reported deviations and the more serious of these should normally be commented upon in the discussion or experimental section of a paper or in the "special_details" fields of the CIF. checkCIF was carefully designed to identify outliers and unusual parameters, but every test has its limitations and alerts that are not important in a particular case may appear. Conversely, the absence of alerts does not guarantee there are no aspects of the results needing attention. It is up to the individual to critically assess their own results and, if necessary, seek expert advice.

Publication of your CIF in IUCr journals

A basic structural check has been run on your CIF. These basic checks will be run on all CIFs submitted for publication in IUCr journals (*Acta Crystallographica*, *Journal of Applied Crystallography*, *Journal of Synchrotron Radiation*); however, if you intend to submit to *Acta Crystallographica Section C* or *E* or *IUCrData*, you should make sure that full publication checks are run on the final version of your CIF prior to submission.

Publication of your CIF in other journals

Please refer to the *Notes for Authors* of the relevant journal for any special instructions relating to CIF submission.

PLATON version of 19/10/2018; check.def file version of 15/10/2018

Datablock Rb4KU7O34F - ellipsoid plot

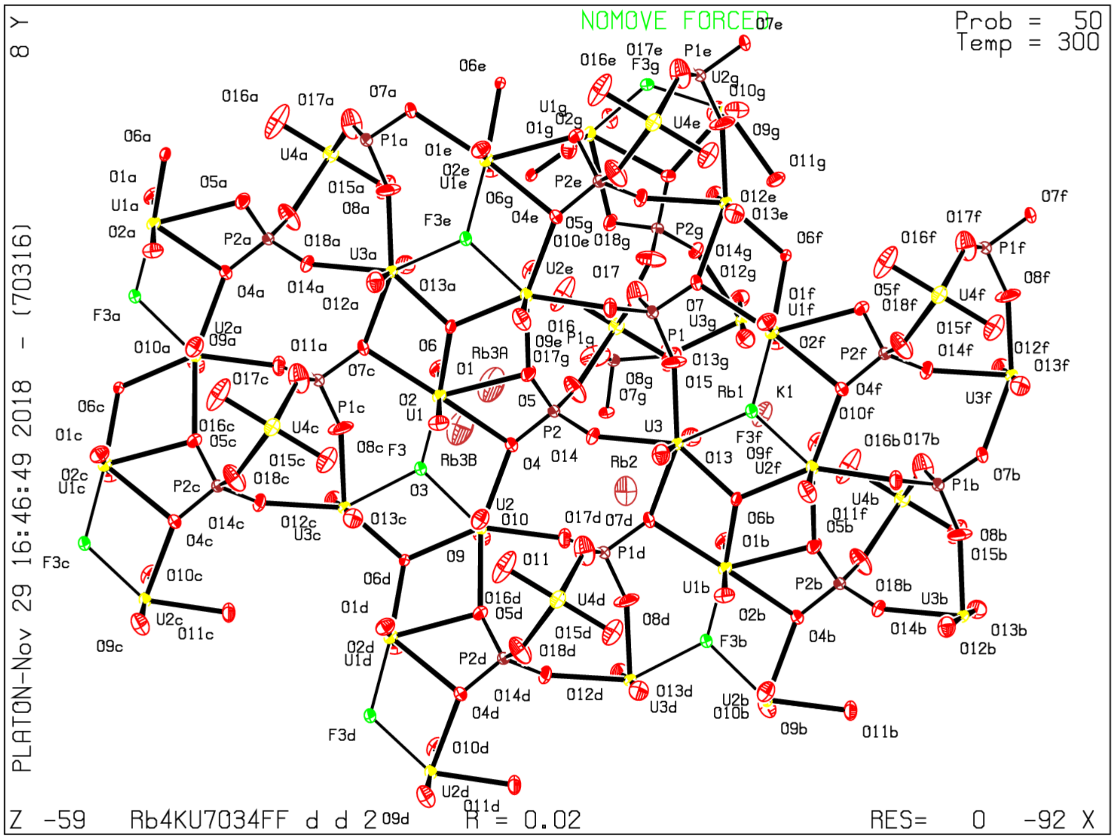

Supplement: Supplementary file 2 [file Data_Sheet_1.PDF]
